# The *Drosophila* Insulin Receptor Independently Modulates Lifespan and Locomotor Senescence

**DOI:** 10.1371/journal.pone.0125312

**Published:** 2015-05-28

**Authors:** Mohd Zamri Bin Haji Ismail, Matt D. Hodges, Michael Boylan, Rajesh Achall, Alan Shirras, Susan J. Broughton

**Affiliations:** Division of Biomedical and Life Sciences, Faculty of Health and Medicine, Lancaster University, Lancaster, LA1 4YQ, United Kingdom; University of Cincinnati, UNITED STATES

## Abstract

The Insulin/IGF-like signalling (IIS) pathway plays an evolutionarily conserved role in ageing. In model organisms reduced IIS extends lifespan and ameliorates some forms of functional senescence. However, little is known about IIS in nervous system ageing and behavioural senescence. To investigate this role in *Drosophila melanogaster*, we measured the effect of reduced IIS on senescence of two locomotor behaviours, negative geotaxis and exploratory walking. Two long-lived fly models with systemic IIS reductions (daGAL4/UAS-InR^DN^ (ubiquitous expression of a dominant negative insulin receptor) and d2GAL/UAS-rpr (ablation of insulin-like peptide producing cells)) showed an amelioration of negative geotaxis senescence similar to that previously reported for the long-lived IIS mutant *chico*. In contrast, exploratory walking in daGAL4/UAS-InR^DN^ and d2GAL/UAS-rpr flies declined with age similarly to controls. To determine the contribution of IIS in the nervous system to these altered senescence patterns and lifespan, the InR^DN^ was targeted to neurons (elavGAL4/UAS-InR^DN^), which resulted in extension of lifespan in females, normal negative geotaxis senescence in males and females, and detrimental effects on age-specific exploratory walking behaviour in males and females. These data indicate that the *Drosophila* insulin receptor independently modulates lifespan and age-specific function of different types of locomotor behaviour. The data suggest that ameliorated negative geotaxis senescence of long-lived flies with systemic IIS reductions is due to ageing related effects of reduced IIS outside the nervous system. The lifespan extension and coincident detrimental or neutral effects on locomotor function with a neuron specific reduction (elavGAL4/UAS-InR^DN^) indicates that reduced IIS is not beneficial to the neural circuitry underlying the behaviours despite increasing lifespan.

## Introduction

The IIS pathway is ubiquitous in multicellular animals [1] and mutations that alter IIS can have pleiotropic effects on growth, development, metabolic homoeostasis and reproduction [2–6]. Despite the potential for severe detrimental effects, such as diabetes in mammals, reduced IIS has been identified as an evolutionarily conserved method of extending lifespan and some measures of age-related health in nematode worms, fruit flies and mice. For example, *Drosophila* lacking *chico*, the single fly insulin receptor substrate are long-lived [7] and show slower age-related decline in negative geotaxis locomotor function [8, 9]. Similarly, mice lacking the insulin receptor substrate 1 (IRS1) are long-lived and show improvements in skin, bone, immune and motor function, and glucose homeostasis with age compared to controls [10]. Although evidence is accumulating that these IIS-related lifespan extending mutations ameliorate some forms of functional senescence, research so far has only scratched the surface in terms of understanding the relationships between IIS, lifespan and healthspan. Indeed, it is becoming clearer that there is a disconnection between functional senescence and lifespan extension due to other genetic or environmental interventions [11–14]. It is thus important to fully evaluate the health and function of long-lived model organisms as they age, to determine if interventions that extend lifespan also have the potential to delay or attenuate ageing-related disease and functional senescence in humans.

In particular, very little is yet known about the effects of lifespan-extending IIS reductions in the central nervous system (CNS) on the behavioural and cognitive senescence that occurs as part of the normal ageing process. IIS plays diverse roles in the CNS and reductions in it can have positive or negative effects on neuronal survival and function [15]. It is possible that IIS reductions may not be beneficial to behavioural health even when they extend lifespan if the positive effects of lowered IIS on peripheral organ systems outweigh any negative or neutral effects in the CNS [15]. Moreover, given the variability in rates and onsets of behavioural declines [16] and the individual sensitivities to IIS of CNS cell types it is likely that manipulating components of this pathway will have diverse effects on the ageing and/or function of the neural circuitries underlying different types of behaviour and cognition. In fact, reductions in IIS have been shown to be detrimental to some behavioural functions. For instance, worms with reduced IIS have been found to show associative learning defects whereas increased IIS improved learning performance [17]. Long-lived *daf-2* mutant worms were found to show improved memory when young and learned better with age, but their long-term memory was not improved at older ages [18]. Similarly, mice with a CNS-restricted deletion of IRS-2 showed a deficit in NMDA receptor-dependent synaptic plasticity in the hippocampus at least at young ages [19]. Interestingly, long lived Ames dwarf mice with reduced circulating levels of IGF-1 showed improved age-related memory retention [20] but this has been suggested to be due to the local production of IGF-1 that occurs in the hippocampus of these mice [21]. Some lifespan extending IIS reductions may therefore result in tissue/cell type specific compensatory increases in insulin-like ligands, which in the CNS may confer protection from potentially negative effects of reduced systemic IIS.

Despite this evidence that reduced IIS can have negative or neutral effects on CNS function and/or behavioural senescence, the potential for beneficial effects has primarily been suggested from studies focussing on the senescence of locomotor behaviour. In *Drosophila*, negative geotaxis (a reflex motor behaviour) has been measured throughout life in long-lived IIS mutant flies. Negative geotaxis is a startle-induced climbing behaviour that is controlled by motor neurons, giant fiber neurons involved in escape behaviour, and possibly other neurons in the CNS [22–24]. The behaviour shows a robust age-related decline (senescence) due to decreases in walking speed [8, 9] that is likely modulated by insulin signalling and other pathways [23–26]. The senescence of this behaviour is ameliorated in long-lived mutant *chico* flies [8] and in flies with overexpression of FOXO in muscle [27], primarily as a result of effects on walking speed with age [9] and improvements in muscle function [27]. In mice, motor function in ageing studies has been assessed by performance in a rotorod test, and long-lived *irs1* ^-/-^ mutant mice show better motor control, coordination and balance than control mice at older ages [10]. The normal performance of any behaviour requires the function of multiple tissues including the CNS, peripheral nervous system and musculature, and it still remains to be determined how reduced IIS in these long-lived animals directly affects CNS ageing and age-specific function.

To directly investigate the role of neural IIS in locomotor senescence, we measured the age-related performance of two different locomotor behaviours—negative geotaxis and exploratory walking—in *Drosophila melanogaster* with ubiquitous or neural-specific IIS reductions. We chose to include an examination of the senescence of exploratory walking because it is a well characterised and complex locomotory behaviour, parameters of which are indicative of decision making processes [28] and thus CNS function. Exploratory walking is a spontaneous activity controlled by the central complex and mushroom bodies of the fly brain [28–30]. These brain structures are involved in the control and regulation of walking parameters such as speed, orientation (direction of walking), bout structure (length and frequency of bouts) and maintenance [28, 30].

We present evidence that supports the hypothesis that long lived IIS mutants display an amelioration of negative geotaxis senescence due to delayed or slowed ageing of peripheral tissues, with IIS playing little part in the neural circuitry controlling negative geotaxis senescence. In contrast, exploratory walking senescence is sensitive to reduced InR activity indicating that reduced IIS can be detrimental to CNS function even when it extends lifespan. Together, the data presented here show that lifespan and the senescence of different locomotor behaviours can be uncoupled, indicating that they are independently modulated by the insulin receptor.

## Materials and Methods

### Fly stocks and maintenance

All fly stocks were initially backcrossed at least 5 times into the white^Dahomey^ outbred background, as previously described [31], and re-backcrossed regularly including just prior to each analysis. daGAL4 and UAS-InR^DN^ are described in [32]; briefly—UAS-dInRA1409K (chr. II) (denoted here as UAS-InR^DN^) was obtained from the Bloomington Drosophila Stock Centre (BDSC); ref. FBal015635). The UAS-InRDN transgene causes an amino acid substitution in the kinase domain (R1409A) of the Drosophila insulin receptor (dInR), resulting in its dominant negative activity. The daGAL4 driver (Daughterless-GAL4) (chr. III) (Fly Base ID FBti0013991)) was obtained from the BDSC and was used for ubiquitous expression of the UAS-InR^DN^ transgene. d2GAL4 and UAS-reaper (UAS-rpr) are described in [3]; briefly—d2GAL4 expresses GAL4 exclusively in the insulin-like peptide producing median neurosecretory cells of the *Drosophila* brain (IPCs) and UAS-rpr expresses the proapoptotic gene reaper. The elavGAL4^C155^ pan-neuronal driver was obtained from the Bloomington stock centre Fly Base ID FBti0002575). Stocks were maintained and experiments conducted at 25°C on a 12h:12h light:dark cycle at constant humidity using standard sugar/yeast medium (100g/L brewer’s yeast (MP Biomedicals), 50g/L sucrose, 10g/L agar)[33]. Flies for all experiments were reared at standard larval density, as previously described [31]. Eclosing adults were collected over a 12 hour period and mated for 48 hours before sorting into single sexes.

### Lifespan

Procedures for lifespan studies are as described in [7]. Lifespan was measured in once mated female or male flies kept at 10/vial on standard food medium and transferred to new food three times a week. Deaths were scored once per day 5–6 times per week.

### Quantitative RT-PCR

*dilp* and UAS-InR^DN^ transcript levels were measured as in [34]. For dilp mRNA analysis primers were: *dilp*2F, TCTGCAGTGAAAAGCTCAACGA; *dilp*2R, TCGGCACCGGGCATG; *dilp*3F, AGAGAACTTTGGACCCCGTGAA; *dilp*3R, TGAACCGAACTATCACTCAACAGTCT; *dilp4*F, GCGGAGCAGTCGTCTAAGGA; *dilp4*R, TCATCCGGCTGCTGTAGCTT; *dilp*5F, GAGGCACCTTGGGCCTATTC; and *dilp*5R, CATGTGGTGAGATTCGGAGCTA; *dilp6*F, CGATGTATTTCCCAACAGTTTCG: *dilp6*R, AAATCGGTTACGTTCTGCAAGTC; *dilp7*F, CAAAAAGAGGACGGGCAATG: *dilp7*R, GCCATCAGGTTCCGTGGTT. Endogenous control primers were as follows: *actin5C*, CACACCAAATCTTACAAAATGTGTGA (forward); and *actin5C*, AATCCGGCCTTGCACATG (reverse). RNA for analysis of UAS-InR^DN^ transgene expression was DNAse I treated prior to cDNA synthesis and primers were: forward GCTGCTGCTGCCATATCGT and reverse GGCAGCAACATGTATCCAG.

### PCR

Single fly preparations and genomic PCR were carried out as previously described [32]. *Wolbachia* primers were designed to amplify a 438bp product from the 16s rRNA gene [35] Primer sequences were: Forward CATACCTATTCGAAGGGATAG and Reverse AGCTTCGAGTGAAACCAATTC.

### Locomotor behavior

**Negative geotaxis** of males and females was measured as described in [36, 37] at weekly intervals throughout the lifespan. Briefly, 15 adult flies were placed in a vertical column (25 cm long, 1.5 cm diameter) and allowed to recover for 30 min. Flies were tapped to the bottom of the column, and flies reaching the top of the column or remaining at the bottom after a 45s period were counted. Three trials were performed at 1 min intervals for each experiment. The mean number of flies at the top (*n*top), the mean number of flies at the bottom (*n*bottom) and the total number of flies assessed (*n*tot) were recorded. Performance index was calculated as 1/2(*n*tot + *n*top −*n*bottom)/*n*tot, as described in [37].

**Exploratory walking** behaviour of individual male or female flies was measured in 4cm diameter/1cm height circular Perspex arenas. Chambers were constructed which contained 4 arenas such that 4 flies could be videoed simultaneously. Individual flies were aspirated into each arena, allowed to rest for one minute and then were videoed for 15 minutes. Videos were analysed using Ethovision XT video tracking software (Noldus), as described in [29]. The walking behavior of 12 flies/genotype/sex was measured in this way at 1, 3, 5 and 7 weeks of age and analysed as described in [8] to calculate total function. All behavioural experiments were carried out at 25°C in parallel with survival analysis of separate cohorts of flies generated and maintained under the same conditions.

### Olfactory avoidance behaviour

The olfactory avoidance assay was performed and performance index calculated as described in Anholt et al (1996). Briefly, vials were marked with 2 lines (3cm and 6cm from bottom). 5 flies were added to a vial placed on its side and a Q-tip dipped in 0.03% v/v benzaldehyde placed into the vial protruding from the cotton wool plug. Flies were allowed to recover for 15 seconds, then the number of flies in the bottom compartment were counted 10 times at 5 second intervals. The avoidance score was calculated as the mean of the number of flies in the bottom compartment over the 10 counts.

### Statistical analyses

Statistical analyses were performed using JMP (version 8) software (SAS Institute). Lifespan data were subjected to survival analysis (Log Rank tests) and presented as survival curves. Other data (QPCR and locomotor behaviour) were tested for normality using the Shapiro-Wilk W test on studentised residuals (Sokal & Rohlf, 1998) and appropriately transformed where necessary. Two-way (genotype and age) or one-way (genotype) analyses of variance (ANOVA) were performed and planned comparisons of means were made using Tukey-Kramer HSD, p<0.05. Data are presented as means of raw data +/- SEM and * denotes significant difference from controls.

## Results

### Systemic lifespan extending reductions in Insulin/IGF-like signalling (IIS) ameliorated the senescence of negative geotaxis locomotory behaviour but neural-specific lifespan extending IIS reductions did not

**Fig 1:**
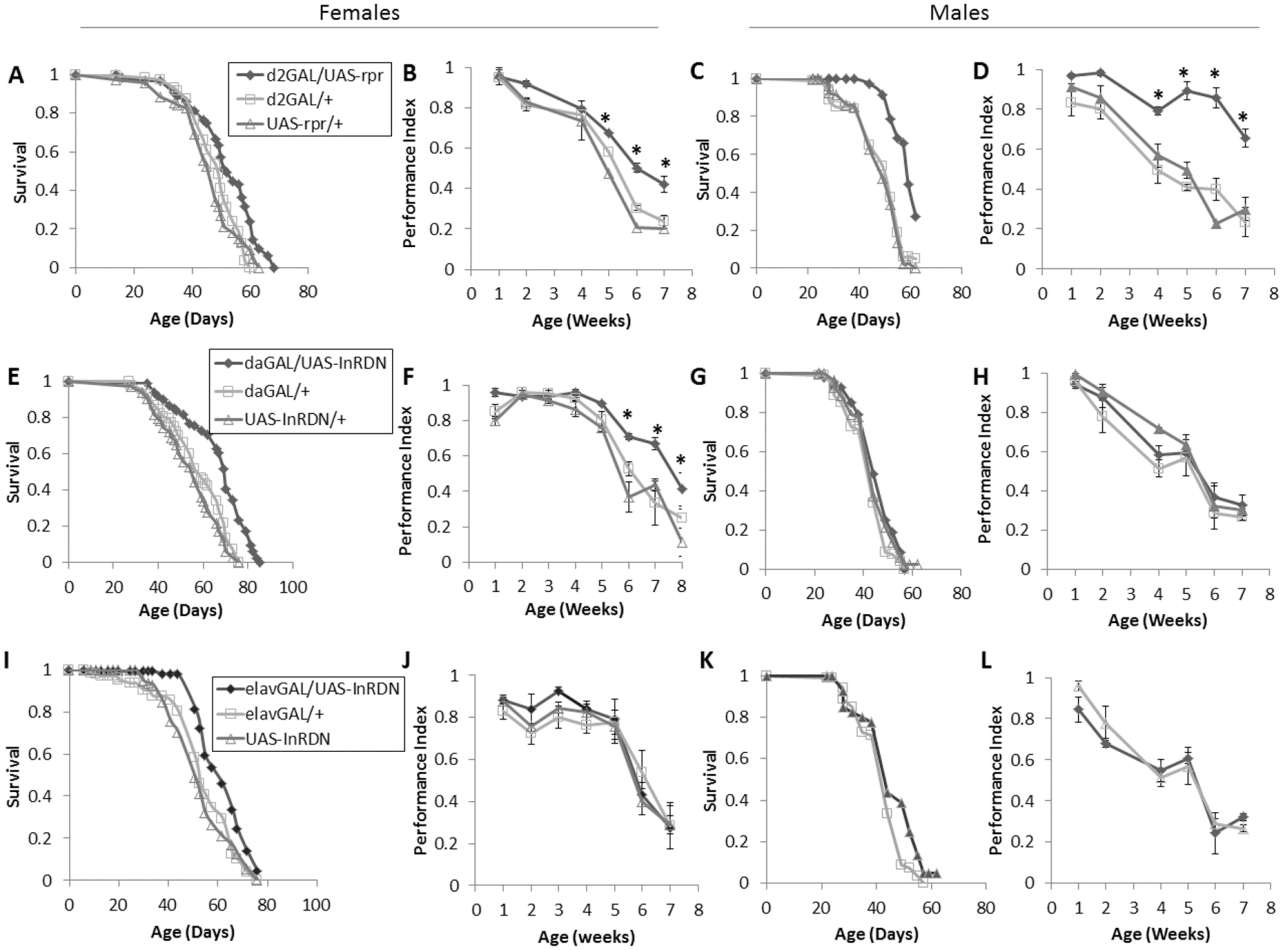
Survival and negative geotaxis senescence in female flies with ubiquitous (d2GAL/UAS-rpr and daGAL4/UAS-InR^DN^) or neuron specific (elavGAL4/UAS-InR^DN^) reductions in IIS. **(A)** Survival of d2GAL/UAS-rpr once mated female flies compared to d2GAL/+ and UAS-rpr/+ controls. Median lifespans and sample sizes were: d2GAL/UAS-rpr = 51 days, N = 71; d2GAL/+ = 49 days, N = 117; and UAS-rpr/+ = 48 days, N = 69. d2GAL/UAS-rpr showed an increased survival compared to both controls by log rank tests (P = 0.001). (**B**) Negative geotaxis performance index (PI) over the lifespan of d2GAL/UAS-rpr once mated female flies compared to d2GAL/+ and UAS-rpr/+ controls, N = 3 (groups of 15 flies) for each genotype. **(C)** Survival of d2GAL/UAS-rpr male flies compared to d2GAL/+ and UAS-rpr/+ controls. Median lifespans and sample sizes were: d2GAL/UAS-rpr = 58 days, N = 70; d2GAL/+ = 47 days, N = 80; and UAS-rpr/+ = 50 days, N = 80. d2GAL/UAS-rpr showed an increased survival compared to both controls by log rank tests (P<0.0001). (**D**) Negative geotaxis performance index (PI) over the lifespan of d2GAL/UAS-rpr male flies compared to d2GAL/+ and UAS-rpr/+ controls, N = 3 (groups of 15 flies) for each genotype. (**E**) Survival of daGAL4/UAS-InR^**DN**^ once mated female flies compared to daGAL4/+ and UAS-InR^**DN**^/+ controls. Median lifespans and sample sizes were: daGAL4/UAS-InR^**DN**^ = 70 days, N = 81; daGAL4/+ = 59 days, N = 84; and UAS-InR^**DN**^/+ = 56 days, N = 97. daGAL4/UAS-InR^**DN**^ showed an increased survival compared to both controls (P<0.0001). (**F**) Negative geotaxis performance index (PI) over the lifespan of daGAL4/UAS-InR^**DN**^ once mated female flies compared to daGAL4/+ and UAS-InR^**DN**^/+ controls, N = 4 (groups of 15 flies) for each genotype. (**G**) Survival of daGAL4/UAS-InR^**DN**^ male flies compared to daGAL4/+ and UAS-InR^**DN**^/+ controls. Median lifespans and sample sizes were: daGAL4/UAS-InR^**DN**^ = 41 days, N = 80; daGAL4/+ = 41 days, N = 80; and UAS-InR^**DN**^/+ = 41 days, N = 80. (**H**) Negative geotaxis performance index (PI) over the lifespan of daGAL4/UAS-InR^**DN**^ male flies compared to daGAL4/+ and UAS-InR^**DN**^/+ controls, N = 3 (groups of 15 flies) for each genotype. (**I**) Survival of elavGAL4/UAS-InR^**DN**^ once mated female flies compared to elavGAL4/+ and UAS-InR^**DN**^/+ controls. Median lifespans and sample sizes were: elavGAL4/UAS-InR^**DN**^ = 60 days, N = 71; elavGAL4/+ = 52 days, N = 84; and UAS-InR^**DN**^/+ = 47.5 days, N = 81. elavGAL4/UAS-InR^**DN**^ showed an increased survival compared to both controls (P<0.0001) (**J**) Negative geotaxis performance index (PI) over the lifespan of elavGAL4/UAS-InR^**DN**^ once mated female flies compared to elavGAL4/+ and UAS-InR^**DN**^/+ controls, N = 3 (groups of 15 flies) for each genotype. (**K**) Survival of elavGAL4/UAS-InR^**DN**^ male flies compared to elavGAL4/+ and UAS-InR^**DN**^/+ controls. Median lifespans and sample sizes were: elavGAL4/UAS-InR^**DN**^ = 56 days, N = 105; elavGAL4/+ = 53 days, N = 89; and UAS-InR^**DN**^/+ = 56 days, N = 95. (**L**) Negative geotaxis performance index (PI) over the lifespan of elavGAL4/UAS-InR^**DN**^ male flies compared to elavGAL4/+ and UAS-InR^**DN**^/+ controls, N = 3 (groups of 15 flies) for each genotype. Negative geotaxis data were analysed by two way ANOVA and age and genotype found to be the main effects (p<0.05). Differences between genotypes at individual time points were analysed by one way ANOVA followed by post hoc means comparisons using Tukey HSD. * indicates significant difference between experimental group and both controls, p<0.05.

To initially determine if the amelioration of locomotor senescence in long-lived *chico* mutant flies is a common feature of lifespan extending systemic IIS reductions, we measured the senescence of negative geotaxis behaviour in two long-lived fly models with systemically reduced IIS—daGAL4/UAS-InR^DN^ flies ubiquitously express a dominant negative form of the insulin receptor under the control of the daughterlessGAL4 driver [32] and d2GAL/UAS-rpr flies have reduced levels of circulating *Drosophila* insulin-like peptides (Dilps) 2, 3 and 5 due to the ablation of insulin producing cells (IPCs) in the brain [31]. These two models were chosen because they are well characterised models of reduced systemic IIS and robustly long-lived [31, 32, 38]. As lifespan extension due to reductions in IIS is dependent on the presence of *Wolbachia* [32, 39], it was confirmed that all strains used in all experiments were positive for this endosymbiont (S1 Fig). As expected, d2GAL/UAS-rpr males and females and daGAL/UAS-InR^DN^ females were long-lived compared to controls (Fig 1A, 1C and 1E), and similarly to long-lived *chico* mutants [8], they all showed an amelioration of negative geotaxis senescence (Fig 1B, 1D and 1F) in line with their extended lifespan. In contrast, daGAL/UAS-InR^DN^ males were normally lived (Fig 1G) as previously observed [32] with a normal senescence of negative geotaxis behaviour (Fig 1H).

To determine the role of neuron-specific IIS in negative geotaxis senescence and lifespan, negative geotaxis and survival were measured in flies expressing the UAS-InR^DN^ transgene under the control of the elav-GAL4 driver (elavGAL/UAS-InR^DN^) which drives expression pan-neurally. Lifespan showed a significant extension in elavGAL/UAS-InR^DN^ female flies compared to controls (Fig 1I) whereas negative geotaxis showed a normal senescence (Fig 1J). Male elavGAL/UAS-InR^DN^ flies showed a normal lifespan and a normal senescence of negative geotaxis behaviour (Fig 1K and 1L).

In summary, negative geotaxis senescence was ameliorated when lifespan was extended by systemic IIS reductions but it was not ameliorated when lifespan was extended by a neuronal IIS reduction. Normally lived flies, irrespective of genotype, showed normal negative geotaxis senescence compared to controls. However, to fully interpret these results we measured UAS-InR^DN^ expression levels in dissected adult brains of elavGAL/UAS-InR^DN^ and daGAL/UAS-InR^DN^ flies by quantitative PCR. Brains of elavGAL/UAS-InR^DN^ flies showed a 5 fold higher level of expression of the UAS-InR^DN^ transgene than daGAL/UAS-InR^DN^, and the very low expression in the daGAL/UAS-InR^DN^ adult brains was similar to that of the UAS-InR^DN^/+ control (S2 Fig). Thus, elavGAL4 drives expression in neurons as expected. However, despite being considered ubiquitous, the daGAL4 driver is expressed at much lower levels in the brain than the neural specific elavGAL4 driver.

Together, these data indicate that the amelioration of negative geotaxis senescence in long-lived flies with systemic IIS reductions is due to effects of IIS in non-neuronal tissues.

### Systemic lifespan extending reductions in IIS had no effect on the senescence of exploratory walking behaviour

**Fig 2:**
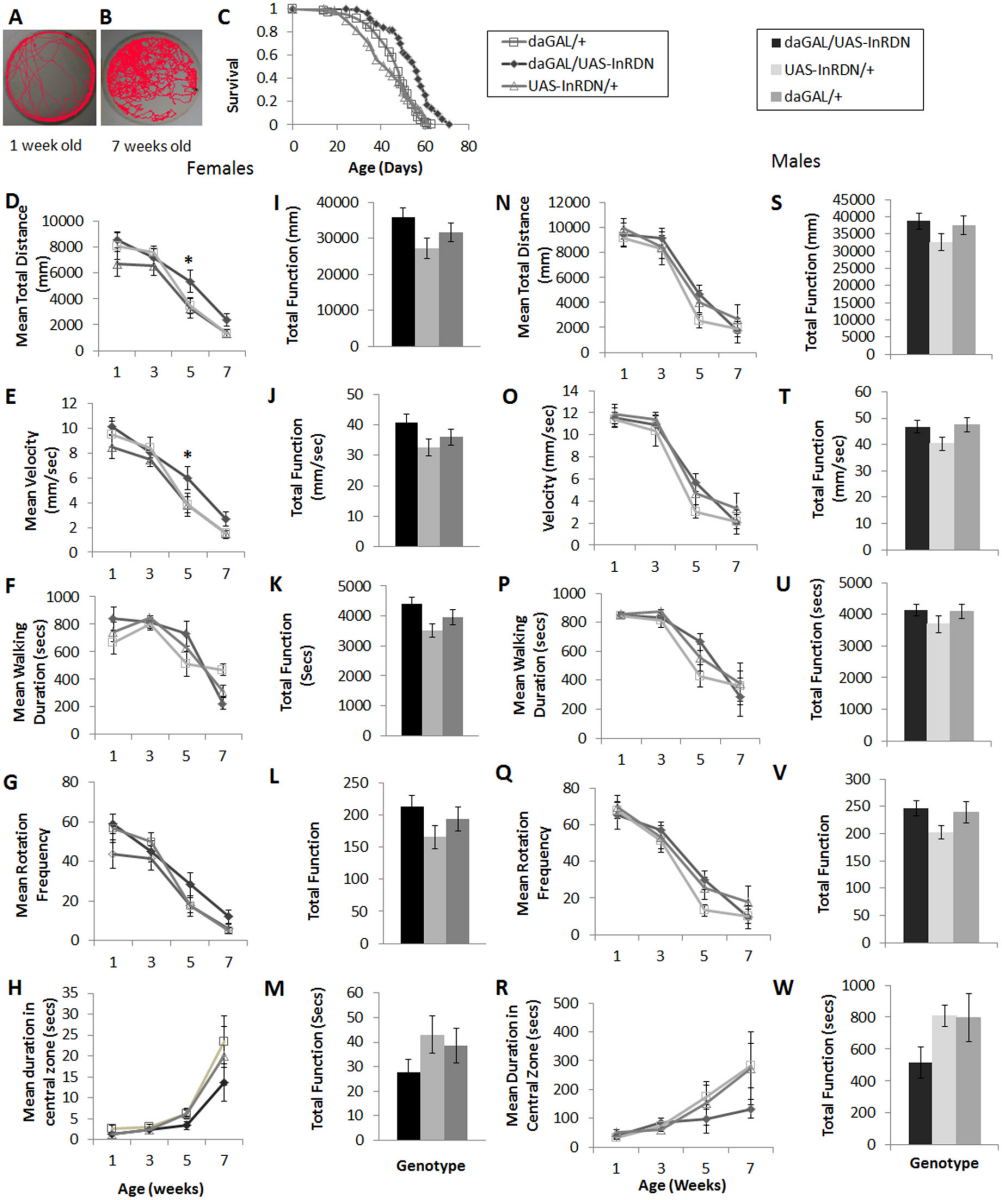
Exploratory walking senescence in daGAL4/UAS-InR^DN^ male and female flies. (**A-B**) Representative images of the exploratory walking track of an individual w^**Dah**^ control female fly at 1 week old (**A**) and 7 weeks old (**B**) during a 15 minute observation period. (**C**) Survival of daGAL4/UAS-InR^**DN**^ once mated female flies compared to daGAL4/+ and UAS-InR^**DN**^/+ controls. Median lifespans and sample sizes were: daGAL4/UAS-InR^**DN**^ = 56 days, N = 69; daGAL4/+ = 48 days, N = 99; and UAS-InR^**DN**^/+ = 44 days, N = 60. Survival curves were compared using nonparametric log rank tests and p values calculated. daGAL4/UAS-InR^**DN**^ showed an increased survival compared to both controls (P<0.0001). (**D-M**) Exploratory walking senescence for a cohort of female flies of the indicated genotypes run in parallel with the survival experiment shown in (C). Data are shown as mean value for each parameter ±SEM, N = 12 for the indicated genotype. (**D**) Female mean distance walked (mm) vs age. (**I**) Female Total Function of mean distance walked (mm). (**E**) Female Mean velocity (mm/sec) vs age. (**J**) Female Total Function of mean velocity (mm/sec). (**F**) Female Mean walking duration (secs) vs age. (**K**) Female Total Function of mean walking duration (secs). (**G**) Female Mean frequency of rotations (change in walking direction) vs age. (**L**) Female Total Function of mean Rotation Frequency. (**H**) Female Mean Duration in Central Zone (secs) vs age. (**M**) Female Total Function of mean duration in central Zone (secs). (**N-W**) Exploratory walking senescence for a cohort of male flies of the indicated genotypes run in parallel with the survival experiment shown in Fig 1G. Data are shown as mean value for each parameter ±SEM, N = 12 for the indicated genotype. (**N**) Male mean distance walked (mm) vs age. (**T**) Male Total Function of mean distance walked (mm). (**O**) Male Mean velocity (mm/sec) vs age. (**T**) Male Total Function of mean velocity (mm/sec). (**P**) Mean walking duration (secs) vs age. (**U**) Male Total Function of mean walking duration (secs). (**Q**) Mean frequency of rotations (change in walking direction) vs age. (**V**) Male Total Function of mean Rotation Frequency. (**R**) Male Mean Duration in Central Zone (secs) vs age. (**W**) Total Function of mean duration in central Zone (secs). Data were analysed by two way ANOVA and genotype and age found to be the main effects (p<0.05). Data at individual time points or total function data were analysed by one way ANOVA followed by post hoc means comparisons using Tukey HSD, and * indicates significant differences (p<0.05) between daGAL4/UAS-InR^**DN**^ flies and both controls.

As the previous data indicated that negative geotaxis senescence is not a sensitive indicator of neural function during normal ageing, we explored the effects of reduced systemic IIS on a different type of locomotor senescence, exploratory walking. This is a complex and spontaneous locomotory behaviour that in young control flies when analysed in a small arena (4cm in diameter) for a short period of time (15 mins) shows a stereotypical pattern of walking bouts, speed and duration of walking, number of rotations (changes in walking direction), and avoidance of the centre of the arena (termed centrophobism) (Fig 2A). Control flies were found to show robust ageing-related changes in these parameters of exploratory walking behaviour, which included decreases in walking speed, distance walked, and frequency of rotations, and increased duration of time spent in the central zone, resulting in old flies (7 weeks) displaying an apparently random pattern of walking (Fig 2B).

**Fig 3:**
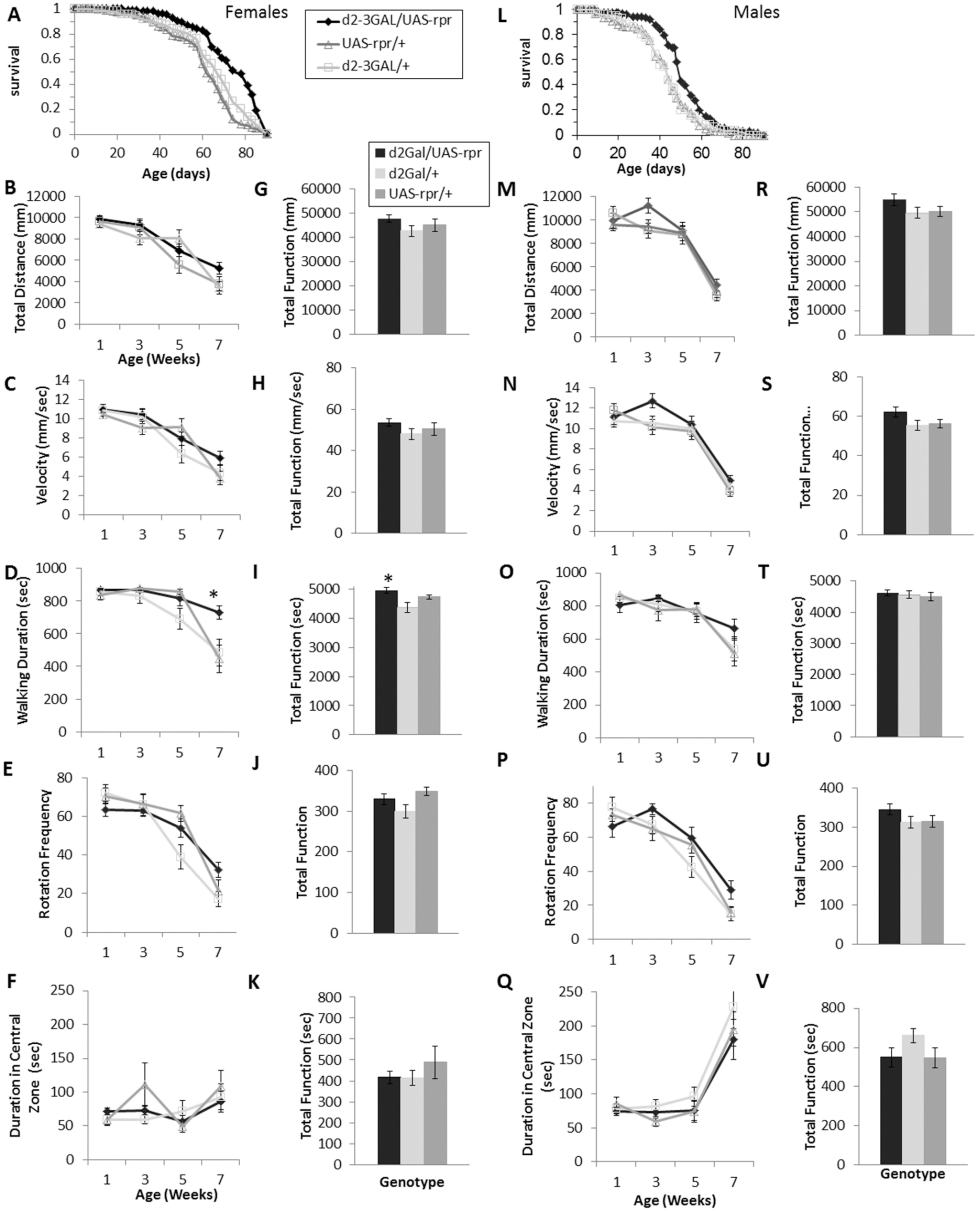
Exploratory walking senescence in long lived d2GAL/UAS-rpr male and female flies. (**A**) Survival of d2GAL/UAS-rpr once mated female flies compared to d2GAL4/+ and UAS-rpr/+ controls. Median lifespans and sample sizes were: d2GAL/UAS-rpr = 76 days, N = 120; d2GAL4/+ = 65 days, N = 120; and UAS-rpr/+ = 61 days, N = 120. Survival curves were compared using nonparametric log rank tests d2GAL/UAS-rpr showed an increased survival compared to both controls (P<0.00001). (**B-K**) Exploratory walking senescence for a cohort of female flies of the indicated genotypes run in parallel with the survival experiment shown in (A). Data are shown as mean value for each parameter ±SEM, N = 12 for the indicated genotype (**B**) Female mean distance walked (mm) vs age. (**G**) Female Total Function of mean distance walked (mm). (**C**) Female Mean velocity (mm/sec) vs age. (**H**) Female Total Function of mean velocity (mm/sec). (**D**) Female Mean walking duration (secs) vs age. (**I**) Female Total Function of mean walking duration (secs). (**E**) Female Mean frequency of rotations (change in walking direction) vs age. (**J**) Female Total Function of mean Rotation Frequency. (**F**) Female Mean Duration in Central Zone (secs) vs age. (**K**) Female Total Function of mean duration in central Zone (secs). (**L**) Survival of d2GAL/UAS-rpr male flies compared to d2GAL4/+ and UAS-rpr/+ controls. Median lifespans and sample sizes were: d2GAL/UAS-rpr = 49 days, N = 120; d2GAL4/+ = 43 days, N = 120; and UAS-rpr/+ = 43 days, N = 120. Survival curves were compared using nonparametric log rank tests and d2GAL/UAS-rpr showed an increased survival compared to both controls (P<0.001). (**M-V**) Exploratory walking senescence for a cohort of male flies of the indicated genotypes run in parallel with the survival experiment shown in (L). Data are shown as mean value for each parameter ±SEM, N = 12 for the indicated genotype. (**M**) Male mean distance walked (mm) vs age. (**R**) Male Total Function of mean distance walked (mm). (**N**) Male Mean velocity (mm/sec) vs age. (**S**) Male Total Function of mean velocity (mm/sec). (**O**) Mean walking duration (secs) vs age. (**T**) Male Total Function of mean walking duration (secs). (**P**) Mean frequency of rotations (change in walking direction) vs age. (**U**) Male Total Function of mean Rotation Frequency. (**Q**) Male Mean Duration in Central Zone (secs) vs age. (**V**) Total Function of mean duration in central Zone (secs). Walking data were analysed by two way ANOVA and age found to be the main effect for all parameters except walking duration where both age and genotype were significant effects (p<0.05). Data at individual time points or total function data were analysed by one way ANOVA followed by post hoc means comparisons using Tukey HSD, and * indicates significant differences (p<0.05) between d2GAL/UAS-rpr flies and both controls.

Exploratory walking was measured in the two long-lived fly models with reduced systemic IIS—daGAL4/UAS-InR^DN^ and d2GAL/UAS-rpr—in two trials. The survival of males and females of each genotype was simultaneously measured to confirm the lifespan effect in each cohort (Fig 2 and 3, S3 Fig and S4 Fig). For daGAL/UAS-InR^DN^ female flies in trial 1, walking speed and distance showed a very small increase at one age point (5 weeks old) compared to controls, although total function over the period of measurement was not significantly increased, and the senescence of other walking parameters was unaffected (Fig 2D–2H). In trial 2, the daGAL4/UAS-InR^DN^ females senesced similarly to controls (S3 Fig). The normally lived daGAL/UAS-InR^DN^ males showed a normal senescence of exploratory walking behaviour in both trials (Fig 2 and S3 Fig). The long lived d2GAL/UAS-rpr females (Fig 3A–3K, S4 Fig) and males (Fig 3L–3V, S4 Fig) showed a normal senescence of all exploratory walking parameters in both trials, except for a very small improvement in walking duration at one age point (7 weeks old) in d2GAL/UAS-rpr females in trial 1 (Fig 3). Thus, systemic IIS reductions have little effect on the senescence of exploratory walking behaviour despite significantly extending lifespan.

### A neuron-specific reduction in IIS deleteriously affected the performance of exploratory walking

The role of IIS in the nervous system in the senescence of exploratory walking behaviour was investigated by measuring lifespan and walking senescence in male and female elavGAL/UAS-InR^DN^ and control flies.

**Fig 4:**
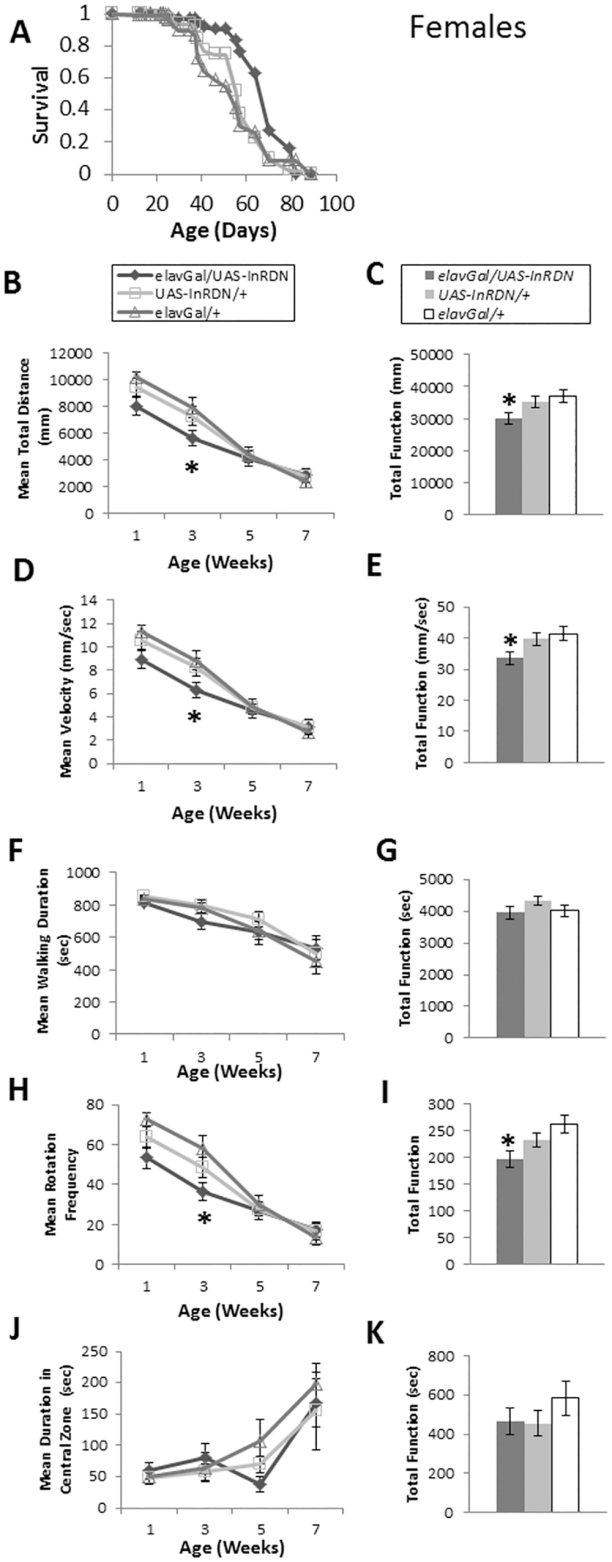
Exploratory walking senescence in elavGAL4/UAS-InR^DN^ female flies. (A) Survival of elavGAL4/UAS-InR^**DN**^ once mated female flies compared to elavGAL4/+ and UAS-InR^**DN**^/+ controls. Survival curves were compared using nonparametric log rank tests and p values calculated. Median lifespans and sample sizes were: elavGAL4/UAS-InR^**DN**^ = 75 days, N = 123; elavGAL4/+ = 61 days, N = 99; and UAS-InR^**DN**^/+ = 56 days, N = 124. elavGAL4/UAS-InR^**DN**^ females showed an increased survival compared to both controls (Log Rank test, p<0.0001). (**B-K**) Exploratory walking senescence for a cohort of female flies of the indicated genotypes run in parallel with the survival experiment shown in (A). Data are shown as mean value for each parameter ±SEM, and N = 15 for the indicated genotype. (**B**) Mean distance walked (mm) vs age. (**C**) Total Function of mean distance walked (mm). (**D**) Mean velocity (mm/sec) vs age. (**E**) Total Function of mean velocity (mm/sec). (**F**) Mean walking duration (secs) vs age. (**G**) Total Function of mean walking duration (secs). (**H**) Mean frequency of rotations (change in walking direction) vs age. (**I**) Total Function of mean Rotation Frequency. (**J**) Mean Duration in Central Zone (secs) vs age. (**K**) Total Function of mean duration in central Zone (secs). Walking data were analysed by two way ANOVA and genotype and age found to be the main effects (p<0.05). Data at individual time points or total function data were analysed by one way ANOVA followed by post hoc means comparisons using Tukey HSD, and * indicates significant differences (p<0.05) between elavGAL4/UAS-InR^**DN**^ flies and both controls.

**Fig 5:**
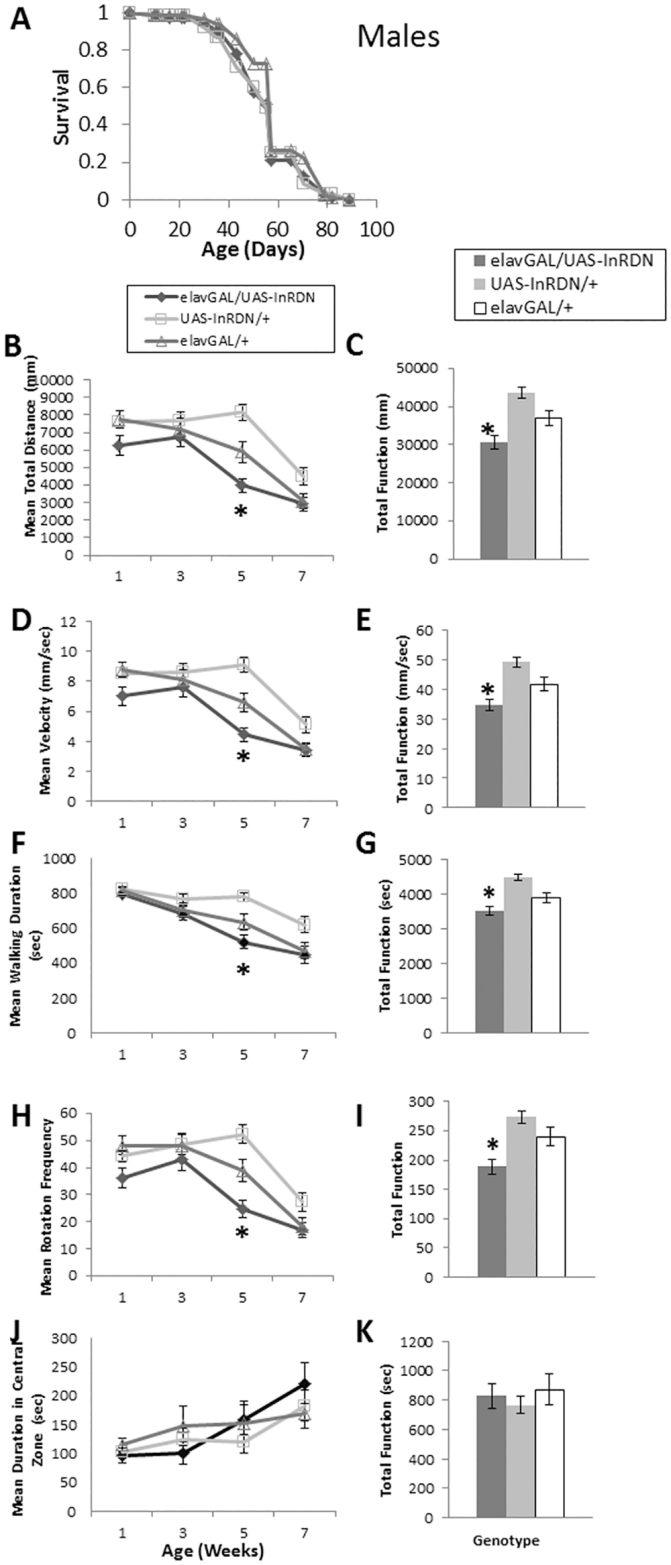
Exploratory walking senescence in elavGAL4/UAS-InR^DN^ male flies. (A) Survival of elavGAL4/UAS-InR^**DN**^ male flies compared to elavGAL4/+ and UAS-InR^**DN**^/+ controls. Survival curves were compared using nonparametric log rank tests and p values calculated. Median lifespans and sample sizes were: elavGAL4/UAS-InR^**DN**^ = 56 days, N = 105; elavGAL4/+ = 53 days, N = 89; and UAS-InR^**DN**^/+ = 56 days, N = 95. elavGAL4/UAS-InR^**DN**^ showed no difference in survival compared to both controls (Log Rank test, p>0.05). (**B-K**) Exploratory walking senescence for a cohort of male flies of the indicated genotypes run in parallel with the survival experiment shown in (A). Data are shown as mean value for each parameter ±SEM, and N = 30 for the indicated genotype. (**B**) Mean distance walked (mm) vs age. (**C**) Total Function of mean distance walked (mm). (**D**) Mean velocity (mm/sec) vs age. (**E**) Total Function of mean velocity (mm/sec). (**F**) Mean walking duration (secs) vs age. (**G**) Total Function of mean walking duration (secs). (**H**) Mean frequency of rotations (change in walking direction) vs age. (**I**) Total Function of mean Rotation Frequency. (**J**) Mean Duration in Central Zone (secs) vs age. (**K**) Total Function of mean duration in central Zone (secs Walking data were analysed by two way ANOVA and genotype and age found to be the main effects (p<0.05). Data at individual time points or total function data were analysed by one way ANOVA followed by post hoc means comparisons using Tukey HSD, and * indicates significant differences (p<0.05) between elavGAL4/UAS-InR^**DN**^ flies and both controls.

Reduced IIS in neurons in elavGAL/UAS-InR^DN^ female flies extended lifespan (as seen previously), and did not ameliorate the senescence of exploratory walking in three independent trials (Fig 4, S5 Fig and S6 Fig). Deleterious age-specific effects on some parameters of exploratory walking behaviour were seen in the three independent experiments, although the magnitude of these negative effects varied across the trials (Fig 4, S5 Fig and S6 Fig). Male elavGAL/UAS-InR^DN^ flies were found to be normally lived (as seen previously) with deleterious age-specific effects on exploratory walking parameters in two independent experiments (Fig 5 and S6 Fig). In addition, although the performance of the behaviour in elavGAL/UAS-InR^DN^ flies at young age showed no significant differences to controls, small reductions in function at 1 week old in two out of three trials in females and one out of two trials in males were seen suggesting a possible functional requirement for the InR in neurons.

The influence of InR^DN^ expression on the function and senescence of sensory systems that may influence exploratory walking behaviour was also considered because sensory input has been found to influence other locomotor behaviours [40]. We measured olfactory avoidance behaviour throughout life in females and found that the elavGAL4/UAS-InR^DN^ flies performed similarly to controls at young ages and showed the same age-related decline as control genotypes (S7 Fig).

Together, these data show that although reducing neuronal InR function is sufficient to extend lifespan in females, it does not ameliorate locomotor senescence in females or males.

### Reduced IIS in neurons extends lifespan in females but not males

**Fig 6:**
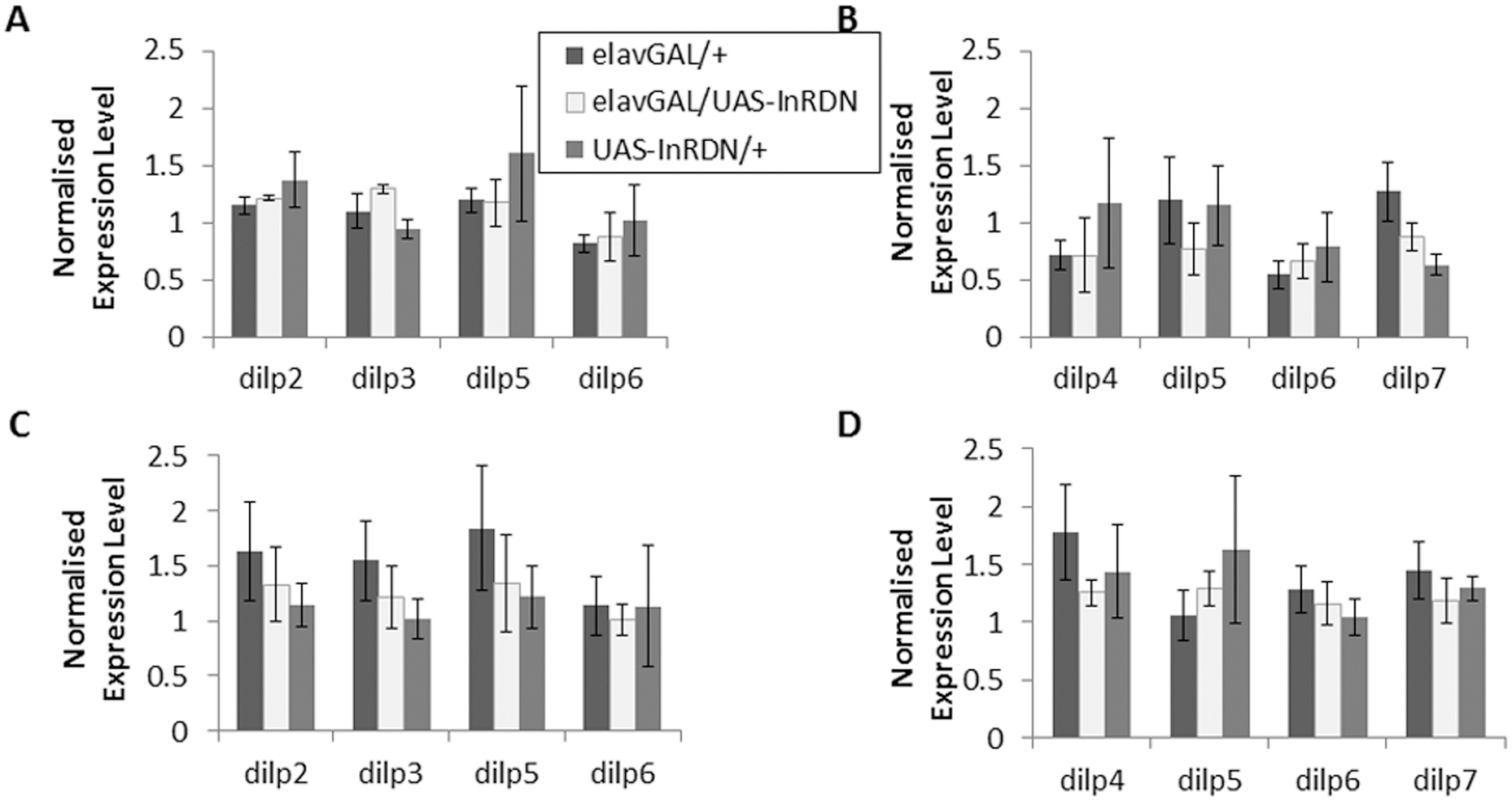
The Effect of InR^DN^ Expression on *dilp* Expression. **(A-D)** The effect of UAS-InR^**DN**^ expression in CNS neurons on *dilp* expression in 10 day old adult male and female heads and bodies was measured by quantitative RT-PCR, and N = 3 (groups of 20 heads or bodies) for each genotype. (**A**) *dilp 2*, *3*, *5* and *6* relative transcript levels in elavGAL/UAS-InR^**DN**^ and control female heads. (**B**) *dilp 4*, *5*, *6* and *7* relative transcript levels in elavGAL/UAS-InR^**DN**^ and control female bodies. (**C**) *dilp 2*, *3*, *5* and *6* relative transcript levels in elavGAL/UAS-InR^**DN**^ and control male heads. (**D**) *dilp 4*, *5*, *6* and *7* relative transcript levels in elavGAL/UAS-InR^**DN**^ and control male bodies. Data are shown as mean relative expression level ±SEM (N = 3).

Measures of survival carried out in parallel with the previous behavioural experiments showed that expression of InR^DN^ in neurons had a sex dependent effect on lifespan. The sexually dimorphic effect seen here is similar to that seen with ubiquitous IIS reductions: males often show a smaller or no lifespan extension due to reduced systemic IIS [31, 32]. To attempt to identify a mechanism in the present study mediating the effect of neuronal InR^DN^ expression on female lifespan, *Drosophila* insulin like peptide (*dilps*) transcript levels were measured in fly heads and bodies of 10 day old adult males and females (Fig 6). The levels of *dilp 2*, *3*, *5* and *6* transcript in adult heads and *dilp 4*, *5*, *6* and *7* in adult bodies were unaffected by InR^DN^ expression suggesting that the lifespan extension was not due to regulation of *dilp* transcription.

## Discussion

To address our current lack of understanding of the tissue specific effects of lifespan-extending IIS reductions on health and functional senescence we manipulated insulin signalling either ubiquitously or specifically in the nervous system of *Drosophila melanogaster*. We measured the effects of reduced InR function on two different types of locomotor functional senescence and on survival, and found that lifespan extension can occur concurrently with normal, ameliorated or reduced locomotor function. That this uncoupling of lifespan and locomotor senescence depends on the type of behavioural function being measured underscores the importance of measuring multiple forms of functional senescence in ageing studies.

Ageing studies in mice and flies have focused heavily on locomotor senescence as a measure of functional healthspan in long-lived animals [8–10, 25]. Lifespan extending IIS reductions in flies have been found to ameliorate the senescence of one type of locomotor senescence, negative geotaxis, due to effects on walking speed [9, 10] via improved muscle function [27]. Although the CNS is known to play a role in controlling this locomotor behaviour [28], the contribution of the CNS to its ameliorated senescence in long-lived IIS mutants had not been elucidated. Therefore, to determine the role of neural IIS in the senescence of locomotor behaviour we targeted a truncated insulin receptor (UAS-InR^DN^) to neurons and measured the senescence of negative geotaxis and exploratory walking. We included an examination of the senescence of exploratory walking because the behaviour encompasses a number of different measurable parameters such as changes in direction and pattern of exploration that are indicative of decision making CNS processes [28, 30] rather than neuromuscular function affecting locomotor speed. Moreover, exploratory walking is complex but easy to measure [29], and we found that it shows a robust, reproducible and stereotypical pattern of senescence in normal flies (Fig 2). We addressed the potential influence of the senescence of sensory systems that may influence exploratory walking behaviour by measuring olfactory avoidance behaviour. However, vision has also been shown to influence exploratory walking such that a lack of visual cues results in flies showing increases in duration of walking and total distance walked [41]. It is unlikely therefore that the reductions in these walking parameters seen in ageing are due to a variable senescence of visual acuity in elavGAL4/UAS-InR^DN^ flies compared to controls. Vision and olfaction have also been shown to influence the decay to spontaneous levels of initial stimulated activity in response to a novel open arena [24]. To reduce the influence of any differential effects of InR^DN^ expression on this short term stimulated activity we began behavioural analysis after one minute of the fly being placed in the arena. It is thus unlikely that a differential senescence of olfactory or visual acuity played a role in the deleterious age-specific effects on exploratory walking seen in elavGAL4/UAS-InR^DN^ flies.

That negative geotaxis senescence was ameliorated in long-lived flies with ubiquitous IIS reductions (daGAL/UAS-InR^DN^ [32] and d2Gal/UAS-rpr [31]) similarly to long-lived *chico* mutant flies [8] supports the notion that improved reflex locomotor function is a common feature of long-lived IIS mutants. However, the lack of improvement in negative geotaxis senescence with neuron-specific IIS reduction (elavGAL/UAS-InR^DN^), despite an extension of lifespan in females, suggests that reduced IIS in neurons does not play a significant role in the amelioration of negative geotaxis senescence. Alternatively, it is possible that muscles age normally in the long-lived elavGAL/UAS-InR^DN^ female flies and any improved neuronal ageing is insufficient to ameliorate negative geotaxis senescence. However, a contrasting role of reduced IIS in neurons vs non-neuronal tissues is supported by the exploratory walking data. Exploratory walking in the long-lived daGAL/UAS-InR^DN^ and d2GAL/UAS-rpr flies showed a generally normal senescence. The lack of significant effect on exploratory walking senescence in daGAL/UAS-InR^DN^ and d2GAL/UAS-rpr flies could be due to the low level of expression of the daGAL4 driver in the adult brain (S2 Fig) and compensatory increases in other DILPs in the brains of d2GAL/UAS-rpr, IPC ablated flies [15]. Moreover, we observed deleterious age-specific effects on multiple parameters of exploratory walking in both male and female flies with neuron-specific IIS reduction (elavGAL/UAS-InR^DN^). Together, these data strongly support the hypothesis that long lived flies with systemic IIS reductions display an amelioration of negative geotaxis senescence (Fig 1; [8]) due to delayed or slowed ageing of peripheral tissues affecting walking speed with IIS playing little part in the CNS circuitry of negative geotaxis senescence. In fact, a major site of the coordination of ageing via IIS is muscle [42, 43] suggesting this as a tissue that responds positively to reduced IIS to ameliorate the senescence of walking speed. In contrast, the CNS circuitries controlling negative geotaxis and the different exploratory walking behavioural parameters are variably sensitive to reduced InR function. Thus, we show an uncoupling of IIS-related lifespan extension from behavioural senescence indicating that lifespan and the senescence of different locomotor behaviours are independently modulated by the insulin receptor.

The detrimental effects on exploratory walking in elavGAL4/UAS-InR^DN^ flies with an apparently normal performance of the behaviour at young ages suggests that the InR is not required for the performance of the behaviour *per se* but is involved in its age-related decline. It is possible that either InR activity is increasingly required with age to maintain the function of neurons or that reduced InR activity speeds up ageing of the neural circuitries underlying exploratory walking. This possibility that reduced IIS acts to promote ageing of the neurons controlling this behaviour is in contrast to the effect of reduced IIS to delay or slow ageing of peripheral tissues. Interestingly, oxidative damage, including lipid peroxidation, is a major factor implicated in neuronal ageing ([44] for review) and the decrease in circulating IGFs with age in mammals has been suggested to lead to increased oxidative stress in the hippocampus which is alleviated by growth hormone replacement [45]. Moreover, the long-lived Ames dwarf mouse, which has decreased circulating IGF but increased IGF in the hippocampus, shows a similar enhanced antioxidant defence capacity in the hippocampus and the periphery [21]. Thus, it appears that reduced IIS in the periphery and increased IIS in the brain can both result in enhanced antioxidant capacity, suggesting that different IIS related pathways may be involved in the brain and periphery in ageing.

However, an alternative explanation that InR function in neurons is required for performance of exploratory walking is suggested by the data: although the elavGAL4/UAS-InR^DN^ flies show no significant reduction in performance at a young age in each trial, they do show small non-significant reductions at 1 week old in 2 out of 3 female trials and 1 out of 2 male trials, and they show an overall lower function at all ages. It is thus possible that the InR is increasingly required for age-specific neuronal function and its down-regulation outweighs any positive effects of reduced IIS on neuronal ageing. The decline in cognitive function during normal ageing is thought in part to involve changes in plasticity mechanisms including long term potentiation (LTP) and synapse formation, and evidence suggests that it may be distinct from neurodegenerative disease [46]. IIS may mediate effects on CNS age-specific function and behaviour via phosphoinositide 3 kinase (PI3K). In *Drosophila*, PI3K regulates synapse number in adult brain projection neurons, PI3K activation can induce synaptogenesis in aged adult neurons, and PI3K activity is required for synapse maintenance [47]. Thus, IIS clearly affects ageing-related synaptogenesis, and it is likely that different types of cognitive and other behavioural functions may be variably sensitive to changes in IIS with age. However, the question remains as to whether or not neuronal ageing *per se* is affected by genetic or environmental manipulations. Despite our data raising the possibility of enhanced neuronal ageing in elavGAL/UAS-InRDN flies, they do not prove that the ageing of the neurons underlying walking is altered. Due to the small age-specific negative effects on walking behaviour and role of IIS in neuronal function it may be more likely that expression of InR^DN^ has a greater negative effect on neuronal function at older ages that outweighs any positive effects of reduced IIS on the underlying ageing of neurons.

This issue of ageing vs age-specific function effects is an important consideration and a major question for future studies that will likely be complicated by the possibility of different neuronal subtypes and circuits having different functional sensitivities to IIS manipulations. For instance, the data presented here suggest that lifespan and the neural circuitries controlling reflex and spontaneous locomotor behaviours respond differently to panneural expression of InR^DN^. Glutaminergic neurons appear to be particularly sensitive to changes in IIS [48] and the central complex cell types controlling walking behaviour in flies express multiple neuropeptides and neurotransmitters, including glutamate [49, 50]. Glutamate, an excitatory neurotransmitter involved in synaptic plasticity, results in an increase in mitochondrial respiration and ROS levels in post synaptic cells which can trigger cell death at high levels, but at physiological levels can up-regulate DNA repair systems which are thought to be an adaptive cellular stress response pathway that protects neurons ([51] for review). However glutamate inactivates AKT antagonising the neuroprotective role of IIS, and reduced sensitivity to IGF-1 has been suggested to be an additional mode of glutamate-induced cell death [48].

In line with previous studies [52, 53], we found that reduced neuronal IIS extended lifespan. The role of IIS in the CNS in determining lifespan has been principally thought to involve the endocrine control of peripheral IIS levels and/or unknown endocrine factors [53–55]. In the IRS2 brain KO mice, it was suggested that the lifespan extension was due to either a neuroendocrine effect or a neuron-specific protection from the neurotoxic effects of a high fat diet [54, 56]. Here, when IIS was reduced only in neurons, lifespan was extended in females and unaffected in males, a common sexually dimorphic effect of IIS manipulations on ageing and lifespan [31, 32]. The lifespan extension of the elavGAL/UAS-InR^DN^ female flies occurred under standard nutrient conditions and so was unlikely to be due to a protection from high food induced neurotoxic *dilp* levels. Analysis of *dilp* transcript levels in males and females suggested that the mechanism of this lifespan extension was not via a neuroendocrine regulation of *dilp* transcript levels from neurosecretory cells or fat body, although it is possible that DILP protein levels were altered. We should add that we have not ruled out peripheral expression of UAS-InR^DN^ in the elavGAL/UAS-InR^DN^ flies but this is very unlikely due to the specificity of the elavGAL4 driver to the nervous system. Although we have not yet identified a mechanism for the lifespan extension we speculate that InR^DN^ expression in neurons could extend lifespan by systemically improving peripheral ageing via either an IIS dependent or independent neuroendocrine mechanism and it is possible that it may involve *dfoxo*-to other signalling [57]. Whatever the mechanism, lifespan could be extended without amelioration of negative geotaxis senescence in elavGAL/UAS-InR^DN^ flies if detrimental effects of reduced IIS on neurons outweigh any improvements in peripheral tissue ageing. Our data are thus consistent with either IIS or non-IIS systemic effects in elavGAL/UAS-InR^DN^ flies. Extensive further research will be needed to identify the signal(s) and/or neuronal circuitry involved in communicating IIS levels in neurons to modulate peripheral tissue ageing and lifespan.

We have shown that lifespan and behavioural senescence are independently regulated by the *Drosophila* insulin receptor, as lifespan extending reductions in IIS do not ameliorate all forms of locomotor senescence such that lifespan extension can occur in the presence of normalameliorated or reducedbehavioural function. These findings illustrate the need for multiple forms of behavioural health to be measured in ageing studies to fully understand the tissue specific mechanisms involved in the modulation of lifespan and healthspan by IIS and other pathways. We suggest that exploratory walking, which shows a robust and complex senescence phenotype, is an excellent indicator of neural mediated locomotor senescence to include in *Drosophila* ageing studies.

## Supporting Information

S1 Fig *Wolbachia* is present in all fly strains used. Single fly preparations and genomic PCR were carried out as previously described (Ikeya et al, 2009). *Wolbachia* primers were designed to amplify a 438bp product from the 16s rRNA gene (Werren &Windsor, 2000). Primer sequences were: Forward CATACCTATTCGAAGGGATAG and Reverse AGCTTCGAGTGAAACCAATTC. kb, kb ladder (Invitrogen). M and F, male and female single fly genomic DNA. Lane 1, UAS-rpr/+. Lane 2, UAS-InR^DN^/+. Lane 3, elavGAL4/+. Lane4, daGAL4/+. Lane 5, w^Dah^. Lane 7, no template control.
(TIF)

S2 Fig The UAS-InR^DN^ transgene is expressed at higher levels in the elavGAL4/UAS-InR^DN^ brains compared to daGAL4/UAS-InR^DN^ brains. 30 brains were dissected per genotype for each RNA extraction and QPCR analysis (N = 3). The elavGAL4/UAS-InR^DN^ genotype showed a significantly higher level of expression of the UAS-InR^DN^ transgene compared to the daGAL4/UAS-InR^DN^ genotype and the UAS-InR^DN^/+ control (p<0.05, ANOVA and Tukey HSD post hoc test). Expression of the UAS-InR^DN^ transgene was not significantly different between the daGAL4/UAS-InR^DN^ genotype and UAS-InR^DN^/+.
(TIF)

S3 Fig Exploratory walking senescence in daGAL4/UAS-InR^DN^ and control flies (trial 2). (**A**) Survival of daGAL4/UAS-InR^DN^ female flies compared to daGAL4/+ and UAS-InR^DN^/+ controls. Median lifespans and sample sizes were: daGAL4/UAS-InR^DN^ = 64.5 days, N = 92; daGAL4/+ = 54 days, N = 99; and UAS-InR^DN^/+ = 54 days, N = 100. daGAL4/UAS-InR^DN^ females showed an increased survival compared to both controls (Log Rank test, p<0.0001). (**B-F**) Exploratory walking senescence for a cohort of female flies of the indicated genotypes run in parallel with the survival experiment shown in (A). Data are shown as mean value for each parameter ±SEM, and N = 12 for each genotype. (**B**) Mean distance walked (mm) vs age. (**C**) Mean velocity (mm/sec) vs age. (**D**) Mean walking duration (secs) vs age. (**E**) Mean frequency of rotations (change in walking direction) vs age. (**F**) Mean Duration in Central Zone (secs) vs age. (**G**) Survival of daGAL4/UAS-InR^DN^ male flies compared to daGAL4/+ and UAS-InR^DN^/+ controls. Survival curves were compared using nonparametric log rank tests and p values calculated. Median lifespans and sample sizes were: daGAL4/UAS-InR^DN^ = 47 days, N = 94; daGAL4/+ = 47 days, N = 98; and UAS-InR^DN^/+ = 44 days, N = 96. daGAL4/UAS-InR^DN^ showed no difference in survival compared to both controls (Log Rank test, p>0.05). (**H-L**) Exploratory walking senescence for a cohort of male flies of the indicated genotypes run in parallel with the survival experiment shown in (G). Data are shown as mean value for each parameter ±SEM, and N = 8 for each genotype. (**H**) Mean distance walked (mm) vs age. (**I**) Mean velocity (mm/sec) vs age. (**J**) Mean walking duration (secs) vs age. (**K**) Mean frequency of rotations vs age. (**L**) Mean Duration in Central Zone (secs) vs age.
(TIF)

S4 Fig Exploratory walking senescence in d2GAL4/UAS-rpr and control flies (trial 2). (**A**) Survival of d2GAL4/UAS-rpr female flies compared to d2GAL/+ and UAS-rpr/+ controls. Median lifespans and sample sizes were: d2GAL4/UAS-rpr = 65 days, N = 120; d2GAL/+ = 52 days, N = 120; and UAS-rpr/+ = 49 days, N = 120. d2GAL4/UAS-rpr females showed an increased survival compared to both controls (Log Rank test, p<0.0001). (**B-F**) Exploratory walking senescence for a cohort of female flies of the indicated genotypes run in parallel with the survival experiment shown in (A). Data are shown as mean value for each parameter ±SEM, and N = 15 for the indicated genotype. (**B**) Mean distance walked (mm) vs age. (**C**) Mean velocity (mm/sec) vs age. (**D**) Mean walking duration (secs) vs age. (**E**) Mean frequency of rotations (change in walking direction) vs age. (**F**) Mean Duration in Central Zone (secs) vs age. (**G**) Survival of d2GAL4/UAS-rpr male flies compared to d2GAL/+ and UAS-rpr/+ controls. Median lifespans and sample sizes were: d2GAL4/UAS-rpr = 51.5 days, N = 120; d2GAL/+ = 47 days, N = 120; and UAS-rpr/+ = 47 days, N = 120. d2GAL4/UAS-rpr males showed an increased survival compared to both controls (Log Rank test, p<0.05). (**H-L**) Exploratory walking senescence for a cohort of male flies of the indicated genotypes run in parallel with the survival experiment shown in (G). Data are shown as mean value for each parameter ±SEM, and N = 15 for the indicated genotype. (**H**) Mean distance walked (mm) vs age. (**I**) Mean velocity (mm/sec) vs age. (**J**) Mean walking duration (secs) vs age. (**K**) Mean frequency of rotations vs age. (**L**) Mean Duration in Central Zone (secs) vs age.
(TIF)

S5 Fig Exploratory walking senescence in elavGAL4/UAS-InR^DN^ female flies (trial 2). Data are shown as mean value for each parameter ±SEM, and N = 12 for each genotype. (**A**) Mean distance walked (mm) vs age. (**B**) Total Function of mean distance walked (mm). (**C**) Mean velocity (mm/sec) vs age. (**D**) Total Function of mean velocity (mm/sec). (**E**) Mean walking duration (secs) vs age. (**F**) Total Function of mean walking duration (secs). (**G**) Mean frequency of rotations (change in walking direction) vs age. (**H**) Total Function of mean Rotation Frequency. (**I**) Mean Duration in Central Zone (secs) vs age. (**J**) Total Function of mean duration in central Zone (secs). Walking data were analysed by two way ANOVA and genotype and age found to be the main effects (p<0.05). Data at individual time points or total function data were analysed by one way ANOVA followed by post hoc means comparisons using Tukey HSD, and * indicates significant differences (p<0.05) between elavGAL4/UAS-InR^DN^ flies and both controls.
(TIF)

S6 Fig Exploratory walking senescence in elavGAL4/UAS-InR^DN^ female flies (trial 3) and male flies (trial 2). (**A**) Survival of elavGAL4/UAS-InR^DN^ female flies compared to elavGAL4/+ and UAS-InR^DN^/+ controls. Median lifespans and sample sizes were: elavGAL4/UAS-InR^DN^ = 47 days, N = 101; elavGAL4/+ = 36 days, N = 92; and UAS-InR^DN^/+ = 41 days, N = 90. elavGAL4/UAS-InR^DN^ females showed an increased survival compared to both controls (Log Rank test, p<0.05). (**B-F**) Exploratory walking senescence for a cohort of female flies of the indicated genotypes run in parallel with the survival experiment shown in (A). Data are shown as mean value for each parameter ±SEM, and N = 15 for the indicated genotype. (**B**) Mean distance walked (mm) vs age. (**C**) Mean velocity (mm/sec) vs age. (**D**) Mean walking duration (secs) vs age. (**E**) Mean frequency of rotations (change in walking direction) vs age. (**F**) Mean Duration in Central Zone (secs) vs age. (**G**) Survival of elavGAL4/UAS-InR^DN^ male flies compared to elavGAL4/+ and UAS-InR^DN^/+ controls. Survival curves were compared using nonparametric log rank tests and p values calculated. Median lifespans and sample sizes were: elavGAL4/UAS-InR^DN^ = 36 days, N = 85; elavGAL4/+ = 36 days, N = 85; and UAS-InR^DN^/+ = 36 days, N = 86. elavGAL4/UAS-InR^DN^ males showed no difference in survival compared to both controls (Log Rank test, p>0.05). (**H-L**) Exploratory walking senescence for a cohort of male flies of the indicated genotypes run in parallel with the survival experiment shown in (G). Data are shown as mean value for each parameter ±SEM, and N = 15 for the indicated genotype. (**H**) Mean distance walked (mm) vs age. (**I**) Mean velocity (mm/sec) vs age. (**J**) Mean walking duration (secs) vs age. (**K**) Mean frequency of rotations vs age. (**L**) Mean Duration in Central Zone (secs) vs age. Walking data were analysed by two way ANOVA and genotype and age found to be the main effects (p<0.05). Data at individual time points were analysed by one way ANOVA followed by post hoc means comparisons using Tukey HSD, and * indicates significant differences (p<0.05) between elavGAL4/UAS-InR^DN^ flies and both controls.
(TIF)

S7 Fig The performance of female flies of the indicated genotype at weekly time points throughout life in olfactory avoidance behaviour. The olfactory avoidance assay was performed and performance index calculated as described in Anholt et al (1996). Benzaldehyde was used at a concentration of 0.03% v/v.
(TIF)
