# Supplementary figures and images for: The Drosophila Insulin Receptor Independently Modulates Lifespan and Locomotor Senescence
Source: PLoS One. 2015 May 28;10(5):e0125312. doi: 10.1371/journal.pone.0125312 (PMC4447345; doi:10.1371/journal.pone.0125312)

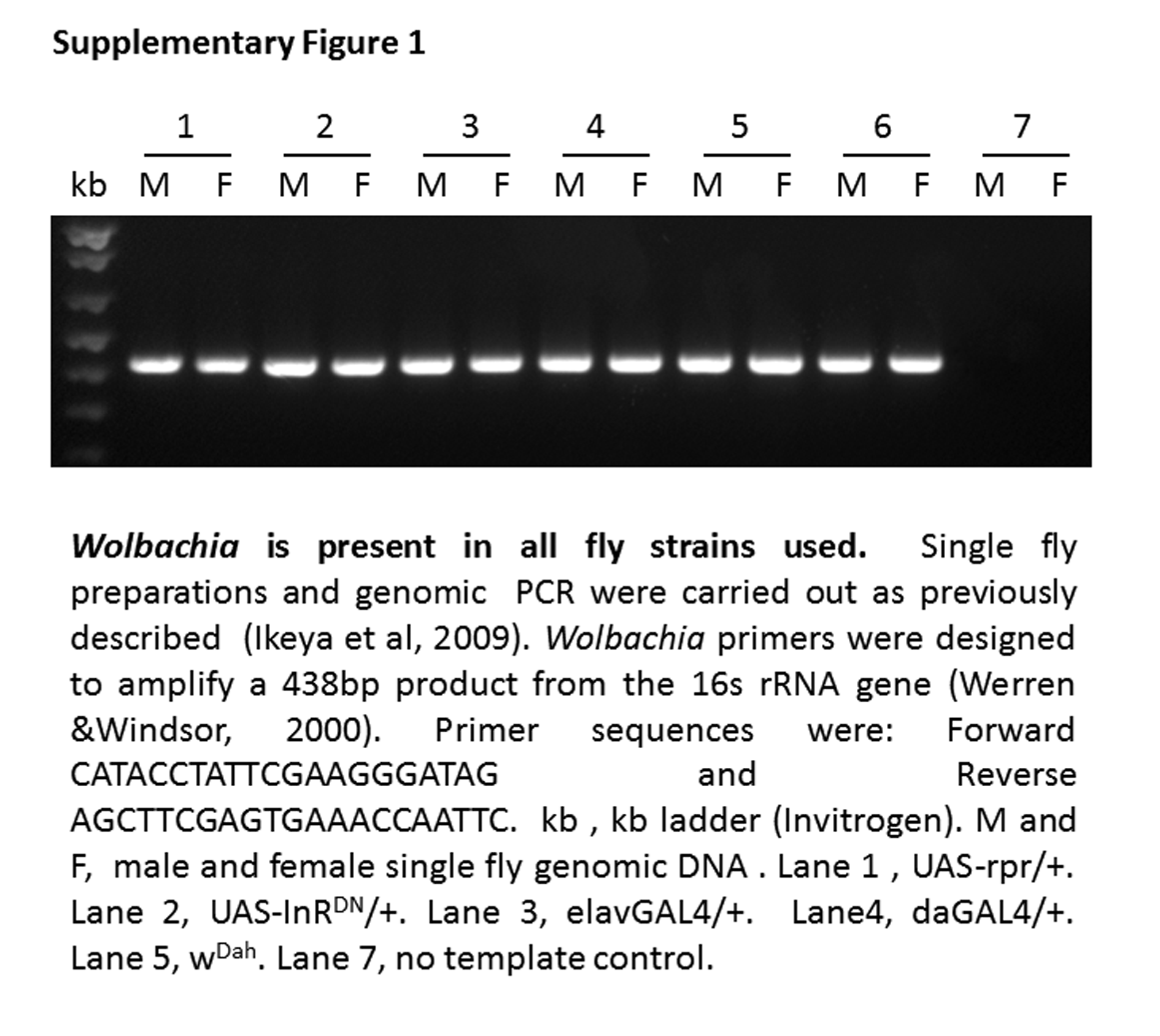

Supplement: S1 Fig — Single fly preparations and genomic PCR were carried out as previously described (Ikeya et al, 2009). Wolbachia primers were designed to amplify a 438bp product from the 16s rRNA gene (Werren &Windsor, 2000). Primer sequences were: Forward CATACCTATTCGAAGGGATAG and Reverse AGCTTCGAGTGAAACCAATTC. kb, kb ladder (Invitrogen). M and F, male and female single fly genomic DNA. Lane 1, UAS-rpr/+. Lane 2, UAS-InRDN/+. Lane 3, elavGAL4/+. Lane4, daGAL4/+. Lane 5, wDah. Lane 7, no template control. (TIF) [file pone.0125312.s001.tif]

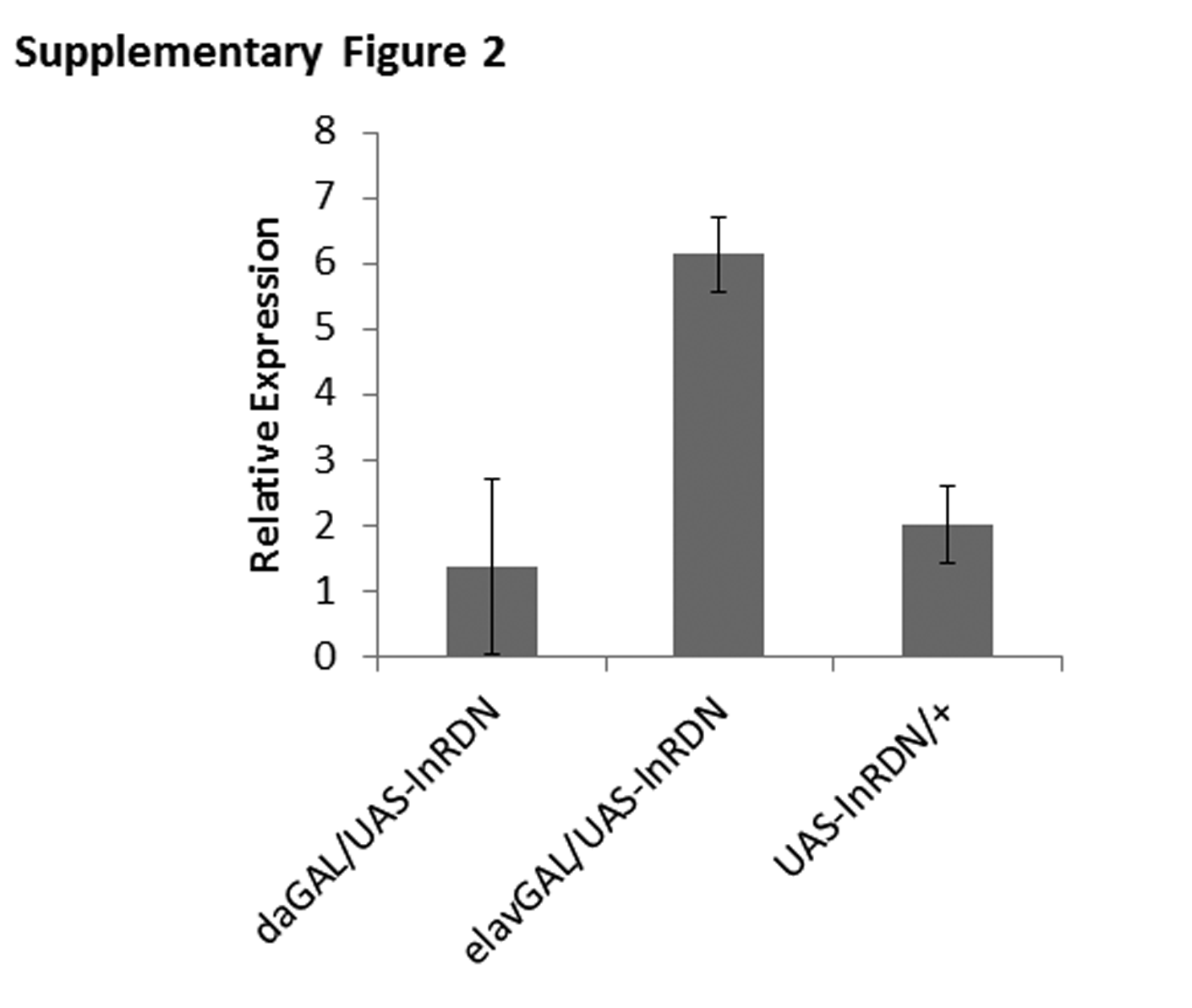

Supplement: S2 Fig — 30 brains were dissected per genotype for each RNA extraction and QPCR analysis (N = 3). The elavGAL4/UAS-InRDN genotype showed a significantly higher level of expression of the UAS-InRDN transgene compared to the daGAL4/UAS-InRDN genotype and the UAS-InRDN/+ control (p<0.05, ANOVA and Tukey HSD post hoc test). Expression of the UAS-InRDN transgene was not significantly different between the daGAL4/UAS-InRDN genotype and UAS-InRDN/+. (TIF) [file pone.0125312.s002.tif]

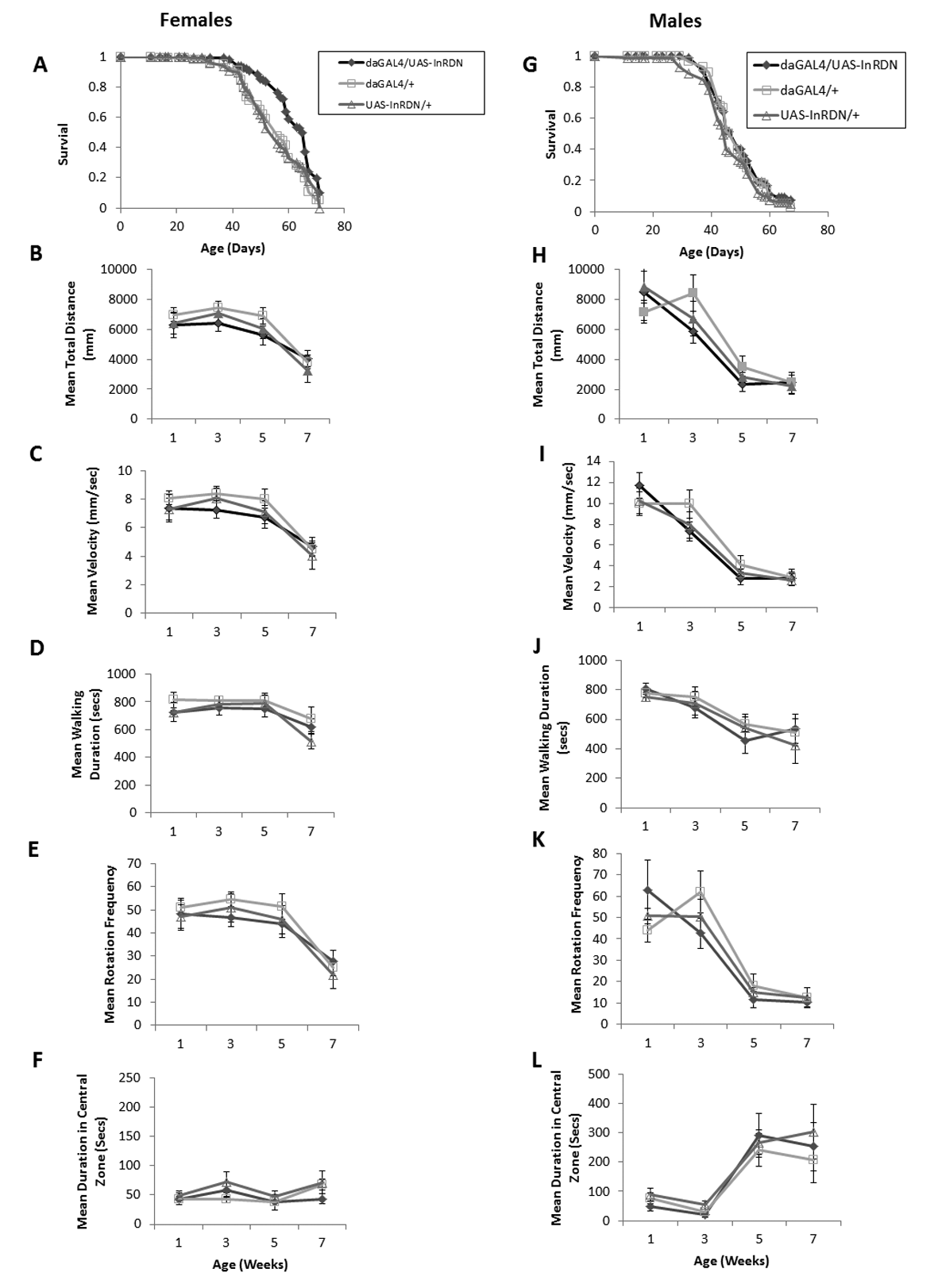

Supplement: S3 Fig — (A) Survival of daGAL4/UAS-InRDN female flies compared to daGAL4/+ and UAS-InRDN/+ controls. Median lifespans and sample sizes were: daGAL4/UAS-InRDN = 64.5 days, N = 92; daGAL4/+ = 54 days, N = 99; and UAS-InRDN/+ = 54 days, N = 100. daGAL4/UAS-InRDN females showed an increased survival compared to both controls (Log Rank test, p<0.0001). (B-F) Exploratory walking senescence for a cohort of female flies of the indicated genotypes run in parallel with the survival experiment shown in (A). Data are shown as mean value for each parameter ±SEM, and N = 12 for each genotype. (B) Mean distance walked (mm) vs age. (C) Mean velocity (mm/sec) vs age. (D) Mean walking duration (secs) vs age. (E) Mean frequency of rotations (change in walking direction) vs age. (F) Mean Duration in Central Zone (secs) vs age. (G) Survival of daGAL4/UAS-InRDN male flies compared to daGAL4/+ and UAS-InRDN/+ controls. Survival curves were compared using nonparametric log rank tests and p values calculated. Median lifespans and sample sizes were: daGAL4/UAS-InRDN = 47 days, N = 94; daGAL4/+ = 47 days, N = 98; and UAS-InRDN/+ = 44 days, N = 96. daGAL4/UAS-InRDN showed no difference in survival compared to both controls (Log Rank test, p>0.05). (H-L) Exploratory walking senescence for a cohort of male flies of the indicated genotypes run in parallel with the survival experiment shown in (G). Data are shown as mean value for each parameter ±SEM, and N = 8 for each genotype. (H) Mean distance walked (mm) vs age. (I) Mean velocity (mm/sec) vs age. (J) Mean walking duration (secs) vs age. (K) Mean frequency of rotations vs age. (L) Mean Duration in Central Zone (secs) vs age. (TIF) [file pone.0125312.s003.tif]

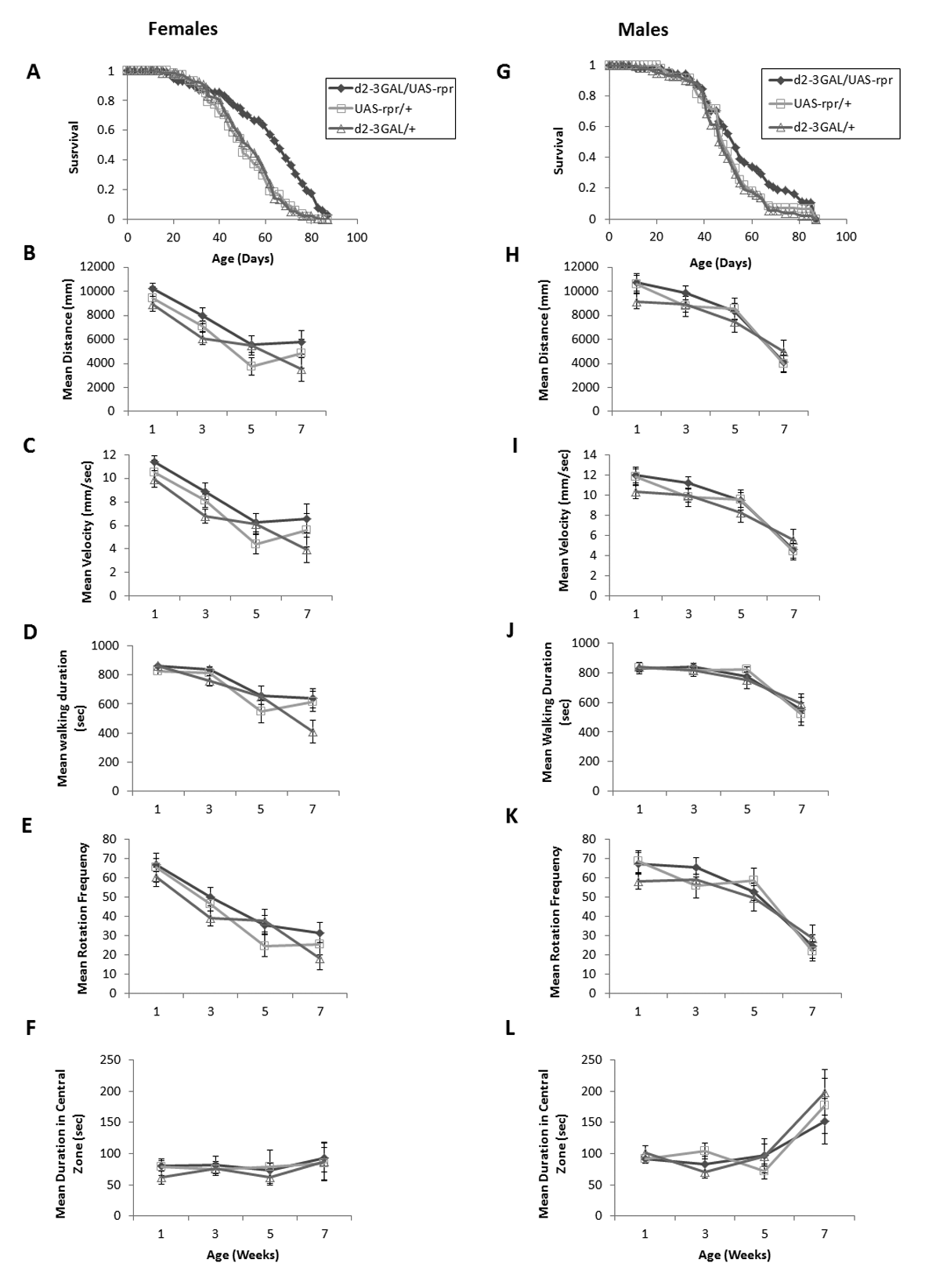

Supplement: S4 Fig — (A) Survival of d2GAL4/UAS-rpr female flies compared to d2GAL/+ and UAS-rpr/+ controls. Median lifespans and sample sizes were: d2GAL4/UAS-rpr = 65 days, N = 120; d2GAL/+ = 52 days, N = 120; and UAS-rpr/+ = 49 days, N = 120. d2GAL4/UAS-rpr females showed an increased survival compared to both controls (Log Rank test, p<0.0001). (B-F) Exploratory walking senescence for a cohort of female flies of the indicated genotypes run in parallel with the survival experiment shown in (A). Data are shown as mean value for each parameter ±SEM, and N = 15 for the indicated genotype. (B) Mean distance walked (mm) vs age. (C) Mean velocity (mm/sec) vs age. (D) Mean walking duration (secs) vs age. (E) Mean frequency of rotations (change in walking direction) vs age. (F) Mean Duration in Central Zone (secs) vs age. (G) Survival of d2GAL4/UAS-rpr male flies compared to d2GAL/+ and UAS-rpr/+ controls. Median lifespans and sample sizes were: d2GAL4/UAS-rpr = 51.5 days, N = 120; d2GAL/+ = 47 days, N = 120; and UAS-rpr/+ = 47 days, N = 120. d2GAL4/UAS-rpr males showed an increased survival compared to both controls (Log Rank test, p<0.05). (H-L) Exploratory walking senescence for a cohort of male flies of the indicated genotypes run in parallel with the survival experiment shown in (G). Data are shown as mean value for each parameter ±SEM, and N = 15 for the indicated genotype. (H) Mean distance walked (mm) vs age. (I) Mean velocity (mm/sec) vs age. (J) Mean walking duration (secs) vs age. (K) Mean frequency of rotations vs age. (L) Mean Duration in Central Zone (secs) vs age. (TIF) [file pone.0125312.s004.tif]

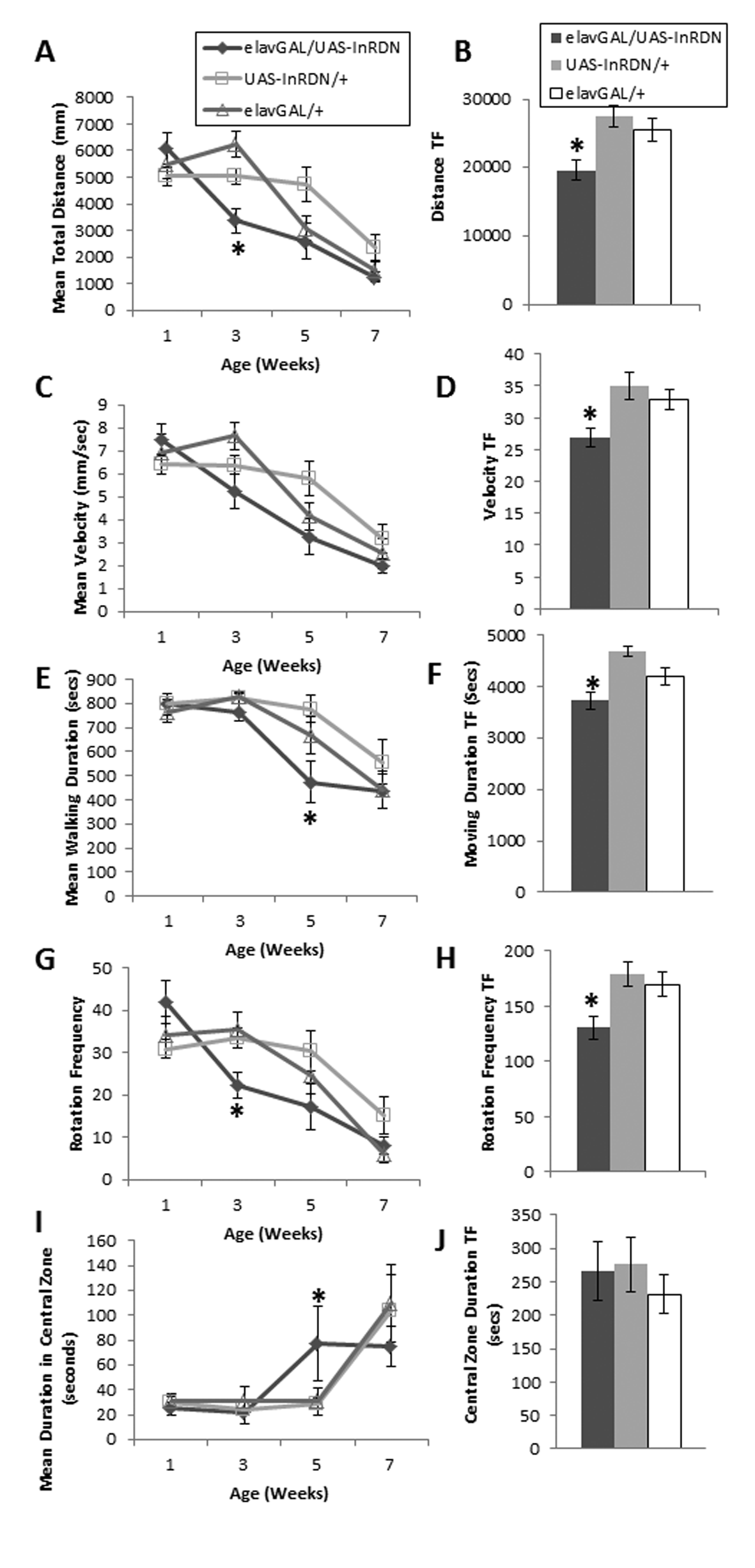

Supplement: S5 Fig — Data are shown as mean value for each parameter ±SEM, and N = 12 for each genotype. (A) Mean distance walked (mm) vs age. (B) Total Function of mean distance walked (mm). (C) Mean velocity (mm/sec) vs age. (D) Total Function of mean velocity (mm/sec). (E) Mean walking duration (secs) vs age. (F) Total Function of mean walking duration (secs). (G) Mean frequency of rotations (change in walking direction) vs age. (H) Total Function of mean Rotation Frequency. (I) Mean Duration in Central Zone (secs) vs age. (J) Total Function of mean duration in central Zone (secs). Walking data were analysed by two way ANOVA and genotype and age found to be the main effects (p<0.05). Data at individual time points or total function data were analysed by one way ANOVA followed by post hoc means comparisons using Tukey HSD, and * indicates significant differences (p<0.05) between elavGAL4/UAS-InRDN flies and both controls. (TIF) [file pone.0125312.s005.tif]

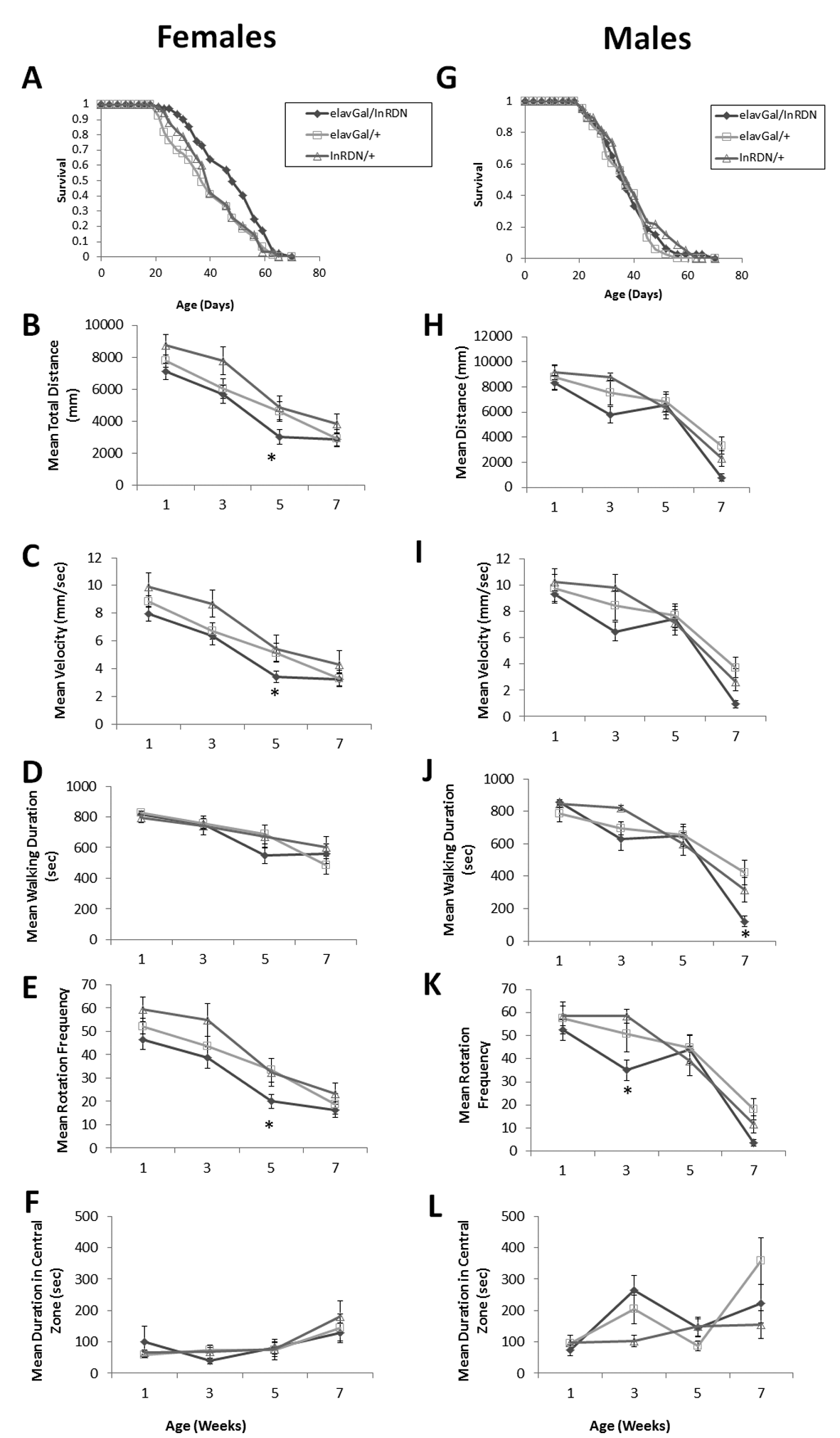

Supplement: S6 Fig — (A) Survival of elavGAL4/UAS-InRDN female flies compared to elavGAL4/+ and UAS-InRDN/+ controls. Median lifespans and sample sizes were: elavGAL4/UAS-InRDN = 47 days, N = 101; elavGAL4/+ = 36 days, N = 92; and UAS-InRDN/+ = 41 days, N = 90. elavGAL4/UAS-InRDN females showed an increased survival compared to both controls (Log Rank test, p<0.05). (B-F) Exploratory walking senescence for a cohort of female flies of the indicated genotypes run in parallel with the survival experiment shown in (A). Data are shown as mean value for each parameter ±SEM, and N = 15 for the indicated genotype. (B) Mean distance walked (mm) vs age. (C) Mean velocity (mm/sec) vs age. (D) Mean walking duration (secs) vs age. (E) Mean frequency of rotations (change in walking direction) vs age. (F) Mean Duration in Central Zone (secs) vs age. (G) Survival of elavGAL4/UAS-InRDN male flies compared to elavGAL4/+ and UAS-InRDN/+ controls. Survival curves were compared using nonparametric log rank tests and p values calculated. Median lifespans and sample sizes were: elavGAL4/UAS-InRDN = 36 days, N = 85; elavGAL4/+ = 36 days, N = 85; and UAS-InRDN/+ = 36 days, N = 86. elavGAL4/UAS-InRDN males showed no difference in survival compared to both controls (Log Rank test, p>0.05). (H-L) Exploratory walking senescence for a cohort of male flies of the indicated genotypes run in parallel with the survival experiment shown in (G). Data are shown as mean value for each parameter ±SEM, and N = 15 for the indicated genotype. (H) Mean distance walked (mm) vs age. (I) Mean velocity (mm/sec) vs age. (J) Mean walking duration (secs) vs age. (K) Mean frequency of rotations vs age. (L) Mean Duration in Central Zone (secs) vs age. Walking data were analysed by two way ANOVA and genotype and age found to be the main effects (p<0.05). Data at individual time points were analysed by one way ANOVA followed by post hoc means comparisons using Tukey HSD, and * indicates significant differences (p<0.05) between elavGAL4/UAS [file pone.0125312.s006.tif]

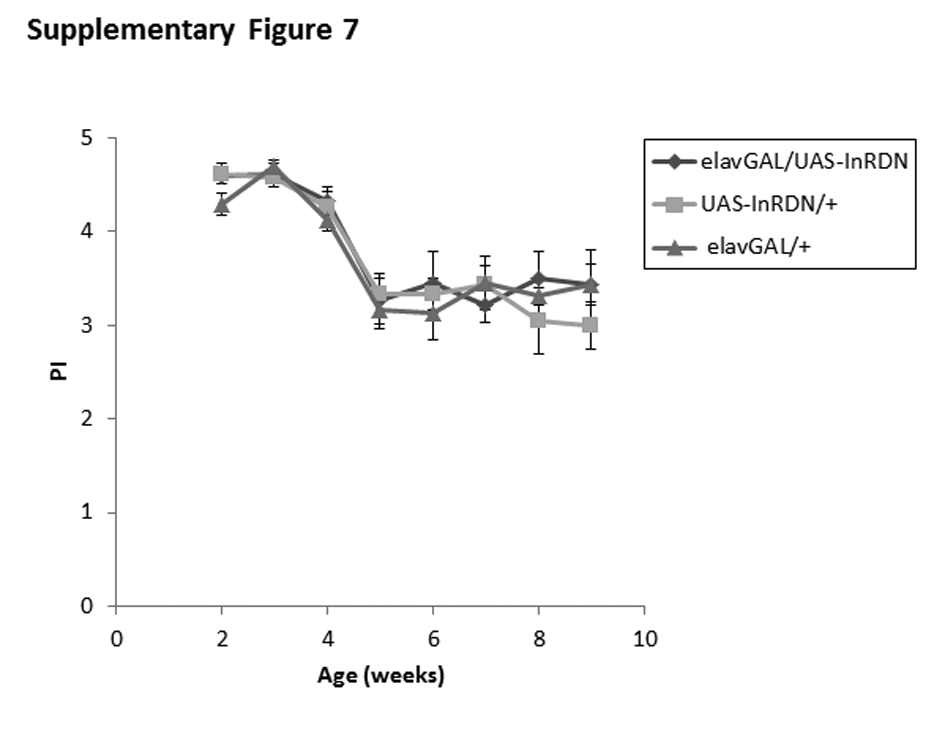

Supplement: S7 Fig — The olfactory avoidance assay was performed and performance index calculated as described in Anholt et al (1996). Benzaldehyde was used at a concentration of 0.03% v/v. (TIF) [file pone.0125312.s007.tif]
